# Quantifying the heterogeneity of macromolecular machines by mass photometry

**DOI:** 10.1038/s41467-020-15642-w

**Published:** 2020-04-14

**Authors:** Adar Sonn-Segev, Katarina Belacic, Tatyana Bodrug, Gavin Young, Ryan T. VanderLinden, Brenda A. Schulman, Johannes Schimpf, Thorsten Friedrich, Phat Vinh Dip, Thomas U. Schwartz, Benedikt Bauer, Jan-Michael Peters, Weston B. Struwe, Justin L. P. Benesch, Nicholas G. Brown, David Haselbach, Philipp Kukura

**Affiliations:** 1 Physical and Theoretical Chemistry Laboratory, Department of Chemistry, University of Oxford, South Parks Road, Oxford, OX1 3QZ UK; 2 Research Institute of Molecular Pathology (IMP), Vienna BioCenter (VBC), Campus-Vienna-Biocenter 1, 1030 Vienna, Austria; 3 Department of Biochemistry and Biophysics and Lineberger Comprehensive Cancer Center, University of North Carolina at Chapel Hill, Chapel Hill, NC 27599 USA; 4 Department of Structural Biology, St. Jude Children’s Research Hospital, Memphis, TN 38105 USA; 5 Howard Hughes Medical Institute, St. Jude Children’s Research Hospital, Memphis, TN 38105 USA; 6 Albert-Ludwigs-Universität, Institut für Biochemie, Albertstr. 21, Chemie-Hochhaus, 79104 Freiburg i. Br., Germany; 7 Department of Biology, Massachusetts Institute of Technology, Cambridge, MA 02139 USA; 8 Department of Pharmacology and Lineberger Comprehensive Cancer Center, University of North Carolina School of Medicine, Chapel Hill, NC 27599 USA; 9 Present Address: Department of Molecular Machines and Signaling, Max Planck Institute of Biochemistry, Martinsried, 82152 Germany

**Keywords:** Structural biology, High-throughput screening, Cryoelectron microscopy, Single-molecule biophysics

## Abstract

Sample purity is central to in vitro studies of protein function and regulation, and to the efficiency and success of structural studies using techniques such as x-ray crystallography and cryo-electron microscopy (cryo-EM). Here, we show that mass photometry (MP) can accurately characterize the heterogeneity of a sample using minimal material with high resolution within a matter of minutes. To benchmark our approach, we use negative stain electron microscopy (nsEM), a popular method for EM sample screening. We include typical workflows developed for structure determination that involve multi-step purification of a multi-subunit ubiquitin ligase and chemical cross-linking steps. When assessing the integrity and stability of large molecular complexes such as the proteasome, we detect and quantify assemblies invisible to nsEM. Our results illustrate the unique advantages of MP over current methods for rapid sample characterization, prioritization and workflow optimization.

## Abstract (web summary)

Mass photometry is a label-free optical approach capable of detecting, imaging and accurately measuring the mass of single biomolecules in solution. Here, the authors demonstrate the potential of mass photometry for quantitatively characterizing sample heterogeneity of purified protein complexes with implications for structural studies specifically and in vitro studies more generally.

## Introduction

Structural and biophysical characterization of biomolecular complexes generally requires the isolation of molecular species from a heterogeneous background of cellular components^1^. The molecules of interest themselves can range from monomeric species to large multimeric machines that undergo conformational rearrangements and form transient interactions with binding partners^2^. As a result, sample heterogeneity remains a significant challenge to the routine and high-throughput structure determination of protein complexes^3^.

Relied-upon methods for protein characterization, including SDS-PAGE, size-exclusion chromatography (SEC) and dynamic light scattering (DLS)^4,5^ report on sample composition but with significant limitations. SDS-PAGE reveals molecular weights, but not stoichiometry or interactions. SEC reports on stokes radii, not the actual molecular weight, and DLS has limited mass accuracy and resolution. A combination of multi-angle light scattering with chromatography (SEC-MALS) is commonly used to determine both Stokes radii and molecular weights simultaneously^6^, but does not operate under equilibrium conditions and heavily depends on the chromatographic resolution achievable and the accuracy of associated protein quantification by UV absorption. Biophysical or structural characterization of protein complexes therefore requires the appropriate combination of these techniques in order to determine and optimize sample quality. Here we describe mass photometry (MP) as an efficient screening tool applicable to a wide range of samples. In particular, we highlight the parallels between MP and a workflow often used in cryo-EM studies for sample optimization where sample quality is assessed using negative-stain EM (nsEM).

For large protein complexes, nsEM is a popular method for evaluating structural integrity and sample composition as it provides a detailed picture of sample heterogeneity under EM conditions while yielding initial structural insights^7^. While the molecular detail that can be extracted is often helpful for further study at high resolution, quantitative data processing and analysis workflows can range from hours to several days, making high-throughput screening impractical. In addition, many samples are poor nsEM candidates or lack prior structural information. To mitigate these limitations, cryoEM-specific approaches have been developed to optimize sample composition and homogeneity^3^, such as variants of the thermofluor technology^8^ and chemical crosslinking combined with density gradient centrifugation^9^.

**Fig. 1:**
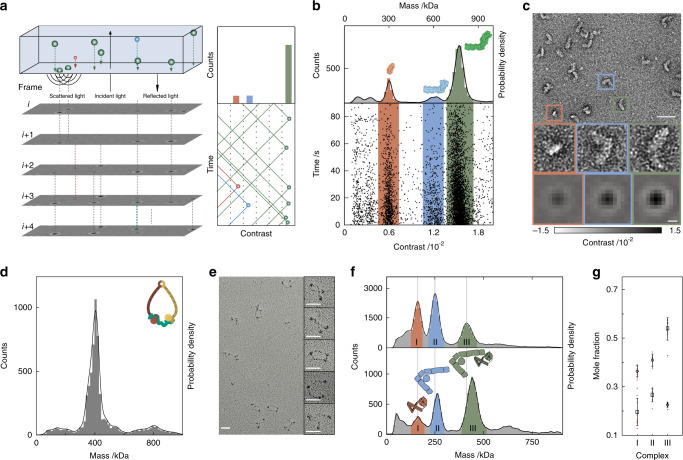
Mass photometry as a general method for characterizing biomolecular heterogeneity. **a** Principle of operation based on interference between scattered and reflected light combined with ratiometric imaging. **b** Scatter plot of binding events for NADH:ubiquinone oxidoreductase (respiratory complex I, 12.5 nM), and the corresponding mass histogram. **c** Negative-stain micrograph of the same sample with individual species corresponding to the peaks in **b** highlighted. MP images of species with the respective mass are shown for comparison. Scale bars: 50 nm (nsEM) and 200 nm (MP). **d** Mass distribution for 21 nM trimeric cohesin; upper right, cartoon of trimeric cohesin. **e** Rotary shadowing EM micrograph of trimeric cohesin shows intrinsic conformational flexibility. Scale bars: 50 nm. **f** A mixture of two interacting NPC-I and II subcomplexes before (top) and after (bottom) chemical crosslinking with 0.1% glutaraldehyde for 5 minutes followed by quenching. **g** Reproducibility of the crosslinking procedure shown in **f** in terms of mole fractions of the three main species before (diamonds) and after crosslinking (rectangles). Source data are provided as a Source Data file.

Oligomeric complexes vary in molecular mass, making mass measurement in principle ideally suited to examine sample heterogeneity. Advances in native mass spectrometry (MS) over the past decades^10–12^ have allowed for much higher mass resolution and can be used for structural analysis, but the associated experimental complexity and non-native conditions have prevented native MS from becoming a widely used tool in this context. Mass photometry (MP), originally introduced as interferometric scattering mass spectrometry (iSCAMS), is a label-free approach that accurately measures molecular mass by quantifying light scattering from individual biomolecules in solution^13,14^. The principle of operation of MP is remarkably similar to the first step of nsEM (Fig. 1a), where placement of a small amount and low concentration (<10 µL, <100 nM) of sample onto a substrate leads to non-specific adsorption at a solid-solute interface. In MP, in contrast to nsEM, a standard microscope cover glass replaces the carbon grid, and no stain needs to be applied.

We then quantify individual binding events by illuminating the interface between the sample and cover glass and interferometrically recording reflectivity changes caused by the change in the local refractive index when an adhering biomolecule replaces water. Continuous recording of these events results in a movie of individual proteins binding to the cover glass surface (Supplementary Movie 1), with species appearing and disappearing in time due to the data analysis procedure^15,16^. Optimization of the image contrast^15^ enables very accurate quantification of the reflectivity change caused by single molecule events, which leads to exceptional mass accuracy, resolution and precision^13,15^ (Supplementary Fig. 1). Here, we show that MP provides quantitative information on the heterogeneity of macromolecular complexes in solution in minutes, and does so using minute sample amounts, under native conditions, and with single molecule sensitivity.

## Results

### Characterization of sample heterogeneity with MP

To first test the applicability of MP in a general case for sample characterization, we chose NADH:ubiquinone oxidoreductase (respiratory complex I) from *Escherichia coli*, a multi-subunit membrane-bound proton pump consisting of six soluble proteins assembled and bound to a large trans-membrane domain of seven different proteins. A scatter plot of single molecule signals arising from a recording of binding events reveals clear bands corresponding to the fully assembled and partially disassembled species (Fig. 1b). We can convert the recorded signals into molecular mass with about 2% mass accuracy by performing a calibration routine with a set of biomolecules of known mass (Supplementary Fig. 2)^13^. The resulting mass distribution shows that the majority of complex I molecules are indeed in the fully assembled state at a complex mass of 770 kDa, in excellent agreement with previous results based on analytical ultracentrifugation^17^. The subcomplex at 600 kDa lacks the substrate acceptor module NuoEFG, while the 300 kDa species corresponds to the hydrophilic portion of the protein only^18^ (Fig. 1b, Supplementary Fig. 3a). A negative-stain micrograph of the same sample qualitatively confirms the variation in recorded masses and heterogeneity of composition (Fig. 1c).

We next sought to examine the limitations of MP when working with flexible proteins by investigating a subcomplex of cohesin containing human SMC1, SMC3, and SCC1 fused to a C-terminal Halo-tag with a predicted molecular weight of 397 kDa. SMC1 and SMC3 consist of long flexible coiled-coils that can switch between a 50 nm extended conformation and a compacted 25 nm conformation. This means that the complex can exhibit conformational as well as mass heterogeneity as a result of disassembly or aggregation^19,20^. The long coiled-coils and the associated structural flexibility make this complex difficult to quantify by conventional nsEM, requiring rotary shadowing EM. This approach confirms the flexibility of the coiled-coils but provides hardly any information on the integrity of the complex. MP, on the other hand, reveals a largely monodisperse distribution centered at 410 kDa, in good agreement with the expected mass and indicates that the sample is of excellent biochemical quality (Fig. 1d,e; Supplementary Fig. 3b). The monodispersity is enabled by the apparent insensitivity of MP signals to large-scale conformational variability on the sub-diffraction length scale, as demonstrated with myosin IIb in our original study^13^.

We then explored whether MP could be used to evaluate sample homogeneity throughout the course of specific sample preparation techniques aimed at stabilizing or enriching specific protein complexes. These approaches are often used to supplement general purification techniques to further optimize sample stability and homogeneity, yet require repeated staining, imaging, and classification when assessed using nsEM. To test whether MP could help alleviate this issue, we studied the interaction between the tetrameric Nup82-Nup145N-Nup159-Nsp1 complex (NPC-I) and a tetrameric Y-complex fragment (NPC-II) from the thermophilic fungus, *Myceliophthora thermophila*. The Y-complex forms the cytoplasmic and nucleoplasmic ring structures of the nuclear pore complex (NPC)^21,22^ and is composed of ~500 individual proteins arranged in subcomplexes around a central eightfold rotational symmetry axis, to facilitate macromolecular transport^23^. We found that NPC-I and NPC-II bind directly and form a 1:1 complex of ~400 kDa (NPC-III), which is to be expected, given that the masses of the individual tetrameric species are 175 kDa and 259 kDa (Fig. 1f, Supplementary Fig. 4). After mild crosslinking with glutaraldehyde, we found clear evidence for enrichment of the 1:1 complex NPC-III. We also observed small increases in molecular mass for NPC-II and NPC-III, as expected due to the addition of crosslinker and quencher. The increases of 11 kDa and 27 kDa are in good agreement with the number of available crosslinking sites and the subsequent quenching procedure (see Methods for full calculation). These results are highly reproducible (Fig. 1g, Supplementary Fig. 5) and enable us to rapidly determine how factors such as incubation time affect the resulting oligomeric distributions and, consequently, sample quality (Supplementary Fig. 6).

### Quantitative comparison of MP with nsEM

**Fig. 2:**
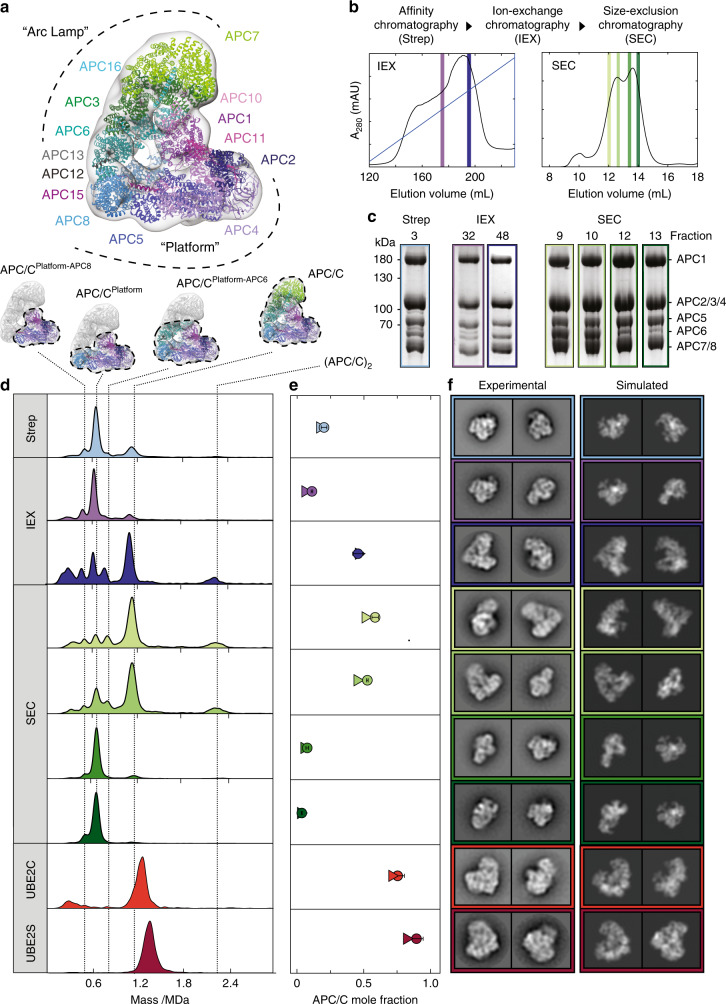
Quantitative comparison of MP with nsEM for anaphase-promoting complex/cyclosome (APC/C) purification and crosslinking. **a** Structural cartoon including highlights of all 14 subunits. **b** Details of the three-step purification protocol and fractions analyzed. **c** SDS-PAGE gels of all fractions highlighted in **b**. **d** Corresponding mass distributions of purification step fractions and APC/C^CDH1^-UBE2C and APC/C^CDH1^-UBE2S traps indicated as UBE2C and UBE2S, respectively. **e** Comparison of assembled fractions obtained by MP (circle) and nsEM (triangle). For the evaluation of mole fractions, we did not consider species below 400 kDa to avoid errors from buffer background for the trap samples. Error bars are calculated as the standard deviation of mole fractions from different repeats. **f** 2D class averages of the two most populated classes obtained by each step are shown along with simulated projections of either APC/C or APC/C^Platform^. Source data are provided as a Source Data file.

To more carefully assess the applicability of MP for characterizing sample quality for cryo-EM, we carried out a side-by-side comparison of the nsEM and MP workflows across an entire purification protocol for the Anaphase-Promoting Complex/Cyclosome (APC/C), a ubiquitin ligase essential for cell cycle progression^24–26^. This large 1.2 MDa scaffold is composed of 19 core polypeptides and transiently associates with numerous binding partners (Fig. 2a, Supplementary Table 1). Purifying homogeneous APC/C scaffolds is thus imperative for detailed biochemical and structural studies. To gain quantitative insights from nsEM, we generated a low-resolution model of both the fully assembled APC/C as well as a subcomplex (termed “Platform”) (Fig. 2a) and projected the resulting maps in-silico (Fig. 2f). We then used these simulated projections as templates for assigning experimentally derived 2D class averages into their corresponding assembly state.

The purification protocol used here involves a three-step scheme: strep-tactin affinity, anion-exchange, and size-exclusion chromatography (Fig. 2b). For fractions from each purification step, we evaluated sample composition by SDS-PAGE (Fig. 2c, Supplementary Figs. 7a,b,c), measured mass distributions by MP (Fig. 2d, Supplementary Figs. 7d, 8, 9), and generated 2D classes using nsEM using the same sample for each step (Supplementary Fig. 10). Strep-tactin affinity chromatography yielded identical SDS-PAGE band patterns and similar mass distributions for each analyzed fraction (Supplementary Fig. 7a,d). The presence of a negligible feature at 1.2 MDa suggests that only a small subset of all species is composed of fully assembled APC/C, while the Platform subcomplex dominates at 660 kDa (Fig. 2d). Quantification of relative mole fractions using nsEM 2D classes confirmed the low fraction of assembled complex observed by MP (17% as measured by nsEM, 21% by MP, Fig. 2e, Supplementary Fig. 11, Supplementary Table 2). The top two 2D classes following strep-tactin affinity chromatography, which are the classes with the highest number of particles out of a set of 200 2D class averages, correspondingly represent subcomplexes rather than the fully assembled APC/C (Fig. 2f). The next purification step, ion-exchange chromatography, resulted in a mixture that also appeared heterogeneous, with the broadened elution peaks providing little information on the underlying distributions (Fig. 2b). SDS-PAGE of the selected fractions confirmed that the key subunits are present but revealed no discernible clues as to sample heterogeneity (Fig. 2c, Supplementary Fig. 7b). The MP distributions from different fractions in the elution profile showed dramatic differences, however, with later fractions containing the highest contribution of fully assembled complexes following ion-exchange (45% for fraction 48, Fig. 2e, Supplementary Fig. 11, Supplementary Table 2). Accordingly, nsEM classification of the later fraction returned fully assembled species for the top two classes, while the earlier fraction consisted mainly of fragments (Fig. 2f). Applying this procedure to different fractions from the size-exclusion chromatography profile demonstrated similar differences between SDS-PAGE and MP and again showed quantitative agreement between MP (9: 60%, 10: 55%) and nsEM (9: 51%, 10: 45%, Fig. 2e, Supplementary Fig. 11, Supplementary Table 2) while also clearly identifying fractions 9 and 10 as optimal for structural analysis.

To evaluate the degree to which sample quality for structural studies can be further optimized using MP to survey crosslinking strategies, we investigated two APC/C complexes that had been previously used for structural studies^27,28^. These samples contained the APC/C scaffold stably bound to a substrate (Hsl1) that is chemically linked to one of its transiently-associated co-factors (UBE2C or UBE2S), hereafter referred to as the “traps” for simplicity. These complexes were purified using tandem affinity chromatography, enriching for both a fully assembled APC/C and the trap, and treated with GraFix^9^, glutaraldehyde crosslinking coupled with density gradient centrifugation, to yield highly purified samples (Fig. 2d, Supplementary Fig. 8). The agreement between MP (APC/C^CDH1^-UBE2S: 89%, APC/C^CDH1^-UBE2C: 75%, Supplementary Fig. 11, Supplementary Table 2) and nsEM (APC/C^CDH1^-UBE2S: 82%, APC/C^CDH1^-UBE2C: 71%) supports our previous findings^27,28^ that these purification strategies optimized sample homogeneity for cryo-EM (Fig. 2e,f).

The slight, but systematic differences observed between the predicted assembled fraction by MP as compared to nsEM could be due to the different ways mole fractions were calculated in MP and nsEM, with MP quantifying only the four (sub-)complexes illustrated in Fig. 2d (See Methods for detailed description), while nsEM picked the full APC/C complex out of all detected particles. At the same time, however, we cannot exclude more fundamental differences due to sample behavior, particularly in negative stain where problems can arise from preferred orientations or the staining process itself.

### Evaluating complex composition, stability, and interactions

**Fig. 3:**
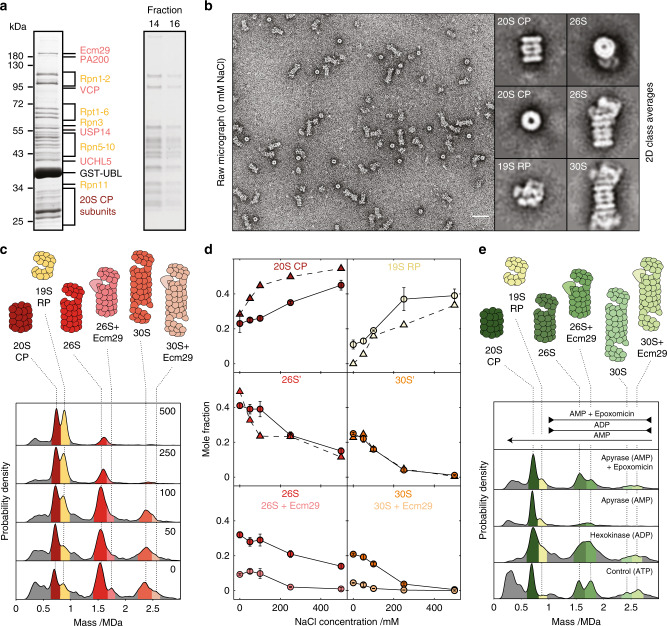
Proteasome composition, structure, stability, and interactions. **a** SDS-PAGE gels of two-step affinity purification of proteasomes—pull down with GST-Ubl (left) and separation on 10–30% sucrose gradient (right). Individual proteasome subunits are shown in dark red and yellow and PIPs in pink. **b** Representative negative-stain micrograph and 2D class averages of proteasome complexes generated by nsEM analysis. Scalebar: 50 nm. **c** MP distributions as a function of NaCl concentration. All reactions were carried out at 4 °C. **d** Corresponding changes to the abundances of the main species comparing nsEM (triangles) with MP (circles) as well as a breakdown of the main 26S and 30S (dark) and Ecm29-bound (light) species. Error bars are calculated as the standard deviation of mole fractions from different repeats. **e** MP distributions as a function of different nucleotide conditions. All reactions were carried out at 37 °C. Source data are provided as a Source Data file.

The purification of protein complexes from their native source using genome editing coupled with affinity purification strategies has become a widely used approach in the light of recent advances enabled by cryoEM. The intrinsic heterogeneity of the resulting complexes, however, yields a plethora of different species in such preparations, in particular in cases involving transient interactions with adapter proteins^29^. To explore a relevant workflow based on buffer composition rather than crosslinking, we studied the stability of the proteasome purified from bovine heart tissue (Fig. 3a, Supplementary Fig. 12). The proteasome itself consists of two subcomplexes: the proteolytically active 20 s core particle (CP) and one or two copies of the ubiquitin recognizing 19 S regulatory particle (RP)^30^ (Fig. 3b, Supplementary Fig. 13a-c, Supplementary Table 3). Our purification approach revealed the presence of adapter protein Ecm29 (~200 kDa), which is thought to assist the CP-RP interaction, of which we could not find any direct evidence in nsEM. The corresponding MP measurements showed the expected features: A 2.4 MDa 30 S particle (2 RP, 1 CP), a 1.5 MDa 26 S particle (1RP and 1 CP), as well as the 700 kDa CP and 800 kDa RP. Additionally, we found signatures of species at both 1.7 MDa and 2.6 MDa, which we assume correspond to the 26 S and 30 S complexes bound to a single copy of Ecm29. Using this sample, we next screened different buffer conditions by altering salt concentrations and nucleotide states. The mass photometry distributions allowed us to observe complex disassembly with increasing salt concentration (Fig. 3c, Supplementary Figs. 14 and 15) as previously reported^31^ and agreed quantitatively with nsEM characterization of the same samples (Fig. 3d, Supplementary Fig. 16, Supplementary Table 4). Interestingly, we found that Ecm29 is the first to dissociate upon salt treatment, in line with previous observations that proteasome interaction proteins (PIPs) are generally salt labile^31,32^.

To further examine the effect of additives, we used a second preparation of bovine proteasomes to assay sample composition in the presence of different nucleotides. We used the enzymes apyrase, which converts ATP to AMP, and hexokinase, which in the presence of glucose converts ATP to ADP. Bringing the samples to 37 °C to ensure mild nucleotide exchange resulted in considerable changes to the distributions of subcomplexes (Fig. 3e, Supplementary Fig. 17). We observed a significant increase in the amount of 26S-bound Ecm29, which agrees with the implication that Ecm29 stabilizes the proteasome upon stress. The persistence of Ecm29 binding upon apyrase treatment despite the almost complete disassembly of pure 26 S implicates its role as a proteasome stabilizer^33^. Furthermore, our data confirms that proteasome disassembly is prevented by the addition of the proteolytic inhibitor epoxomicin (Fig. 3e), most likely through a long-range allosteric effect^33,34^. The observed variability in the distribution of complexes can be confidently assigned to salt and nucleotide-induced effects, given the extreme stability of our proteasome preparations (Supplementary Fig. 18).

## Discussion

Our results demonstrate that MP provides information on sample composition that closely correlates with those obtained by nsEM (Supplementary Fig. 19), but with a number of key advantages. Measurements take place in native conditions, are extremely fast (<1 min), and require minimal sample amounts (<pmole). Assessment of the results does not require prior knowledge of protein structure but instead provides direct information on the sub-assemblies that are present in the sample by revealing the masses of all species. Quantitation by MP closely agrees with data obtained by nsEM, but MP is not subject to the complications that can arise in EM such as stain artifacts, errors in image processing including false particle picking, and alignment or classification issues (Supplementary Fig. 13d), while also characterizing species that are difficult or impossible to visualize by nsEM. In comparison to SEC-MALS, MP provides improved resolution, in situ measurement of mixtures without need for separation by chromatography, and much lower demands in terms of sample volume and concentration. The main limitations of MP, on the other hand, are a lack of structural detail, as well as a currently limited concentration range (<100 nM) due to the single particle nature of the approach. We expect the concentration limitations to be lifted in the near future with improvements in assays, instrumentation, and data analysis. We also emphasize that measurements at lower concentrations are still valuable for two reasons: (1) As long as the associated off-rate is on the order of minutes or slower, the distributions obtained by MP are representative of those at higher concentrations because of the speed of the measurement. (2) Even in the case of faster off-rates, comparison of different samples with MP will be valuable, unless very fast off-rates on the order of seconds are encountered, because complexes will not have completely dissociated prior to measurement. In addition, at this point of development, it is unclear to which degree the overall structure of macromolecules well below the diffraction limit affects their quantification by MP. In our previous work^13^ we could not detect significant effects due to structural changes on the measured mass. Evidence so far suggests that MP is insensitive to conformation within the 5–10% level of measured mass, but there is ample scope for exploration of these details in the future.

Overall, MP will be of tremendous value to the cryo-EM and X-ray crystallography communities not only by significantly improving the efficiency of structure determination, but also enabling studies aimed at understanding (dis)assembly processes through kinetic and reconstitution assays. More generally, the capabilities of MP will likely impact the broader life science community to enable accurate sample characterization for the majority of biochemical and biophysical in vitro studies.

## Methods

### Mass photometry

Microscope coverslips (No. 1.5, 24 × 50 and 24 × 24 mm^2^, VMR) were cleaned by sequential sonication in 50% isopropanol (HPLC grade)/Milli-Q H_2_O, and Milli-Q H_2_O (5 min each), followed by drying with a clean nitrogen stream. Clean coverslips were assembled into flow chambers using double-sided-sticky tape (3 M) as described by Young et al.^13^. Fresh aluminium foil was folded around an A4 size cutting board. Individual 24 × 24 coverslips were taped using two strips of double-sided tape and cut from the foil using a scalpel blade. Each excised 24 × 24 coverslip was joined, tape side down, in the center of a 24 × 50 coverslip and stored prior to use.

Immediately prior to mass photometry measurements, protein stocks were diluted directly in stock buffer (unless stated otherwise). Typical working concentrations of protein complexes were 5–25 nM, depending on the dissociation characteristics of individual assemblies. Each protein was measured in new flow-chambers (i.e., each flow-chamber was used once). To find focus, fresh buffer was first flowed into the chamber, the focal position was identified and secured in place with an autofocus system based on total internal reflection for the entire measurement. For each acquisition, 15 µL of diluted protein was introduced into the flow-chamber and, following autofocus stabilization, movies of either 60 or 90 s duration recorded. Each sample was measured at least three times independently (*n* ≥ 3).

All measurements were performed using similar mass photometry instruments. Most data was acquired using a One^MP^ mass photometer (Refeyn Ltd, Oxford, UK) except for nuclear pore complex (NPC) crosslinking experiments, which were performed on a home-built mass photometer constructed as discussed in detail by Cole et al.^15^, except for operation at the same wavelength as the commercial instrument. Data acquisition was performed using either AcquireMP (Refeyn Ltd, v1.1.3—proteasome measurements and v1.2.1 for all other measurements) or custom software written in Labview (for NPC crosslinking^16^). Mass photometry movies were recorded at 1 kHz, with exposure times varying between 0.6 and 0.9 ms, adjusted to maximize camera counts while avoiding saturation. Images were time averaged 5-fold and pixel binned 4 × 4, before saving. The time and pixel binning resulted in an effective pixel size of 84.4 nm and effective frame rate of 200 Hz.

### Image processing

All MP images were processed and analyzed using DiscoverMP (Refeyn Ltd, v1.2.3). In short, the procedure included three main steps: (1) background removal, (2) identification of landing particles and 3) particle fitting to extract maximum contrast. Background removal—static scattering background from the glass-water interface was removed by calculating the ratiometric images, *R*, as $R_m = N_m/N_{m - 1} - 1$, where $N_m(x,y) = \mathop {\sum}\nolimits_{i = m}^{m + {\mathrm{nf}}} {F_i} (x,y)$ is the sum of each pixel in consecutive images (*F*), with nf defining how many frames to sum^16^. In this way, images in the field of view are preserved, while eliminating any background. This procedure is applied to all possible frames, creating a ratiometric movie (Supplementary Movie 1), where the binding of particles to the glass-water interface is clearly visible. Identification of landing particles—a landing particle generates a step-wise increase in the glass reflectivity which results in an increase in scattering signal, followed by an amplitude decrease ratiometric movie^16^. This distinct signature of step-wise behavior is used to identify particles, using two fitting parameters; threshold 1 (related to the particle contrast relative to the background noise) and threshold 2 (related to the radial symmetry or the particle signature). Particle fitting—identified particles were fit using a model point spread function (PSF) in order to extract the contrast. Supplementary Fig. 1 shows the experimental and fitted PSF with the corresponding residuals, emphasizing the accuracy of the fitting procedure, both for particles with large (top panel) and small (bottom panel) signal-to noise ratio. Our model PSF does not necessarily capture the spatially more extending features of our PSF, which means that the fit residuals are not entirely dominated by shot noise (Supplementary Fig. 1c,f). Based on experience, we find that these differences do not introduce a systematic error in quantification of the signal, as long as the same PSF model is applied for the entire data set and calibration.

Table 1 specifies the values of the fitting parameters for all examined samples in this work. The standard values of binned frames (nf) are between 3 and 5 frames, while threshold 1 (T1) values varies according to the contrast to noise values, with standard values between 0.5 and 1.5. To quantify low mass proteins below 100–200 kDa either lower T1, and\or higher nf values are chosen (examples are calibration proteins). In cases where the samples have noisier backgrounds, usually related to buffer content or high amounts of smaller proteins, T1 is increased to 2 or 3, to avoid detection of low mass signals which are not quantitative in those cases. For APC/C and proteasome samples, the values of nf (3–5) and T1 (2–3.5) are almost equivalent, since changes in those values will affect only the low mass regime (<300 kDa). Therefore, all masses below 300 kDa in APC/C and proteasome samples are not used for the quantitative analysis of mole fractions.

**Table 1:** DiscoverMP analysis parameters for all protein complexes. The values for threshold 2 (0.25) and median filter kernel (15) remained constant for all protein samples.

Protein	Number of binned frames, nf	Threshold 1 (T1)
Respiratory complex I	5	0.5
Trimeric cohesin	5	0.8
NPC	5	0.8
APC/C—affinity chromatography	3	2
APC/C—ion-exchange chromatography	5	3
APC/C—size-exclusion chromatography	3	2
APC/C—crosslinked	3	2
Proteasome	5	3.5

### Calibration procedure

Contrast-to-mass (C2M) calibration was performed daily and for each buffer solution separately, since the C2M conversion may change slightly as a result of buffer content. The calibration protocol included measurement of two protein oligomer solutions, one with masses of 66, 132, and 198 kDa and a second with masses of 90, 180, 360, and 540 kDa. Each MP calibration experiment was analyzed using DiscoverMP. The mean peak contrast was determined in the software using Gaussian fitting. The mean contrast values from both calibration protein solutions were then plotted (Supplementary Fig. 2) and fitted to a line, $y = bx$, with *y*—contrast, *x*—mass and *b*—C2M calibration factor. The errors were calculated from the residuals of measured contrast and calibration curve values, with the mean error extracted from the average of five calibration repeats and error bars representing the standard deviation.

The mass distributions for the two protein oligomer solutions are shown in Supplementary Fig. 2b, c. The achievable precision of the order of 2% of the object mass for repeated measurements, which is intrinsic to mass photometry, is larger than the standard error of the mean $\sigma N^{ - 0.5}$, given that *σ* < 20 kDa throughout the mass range used, and N is >100.

### Extraction of mole fractions

The output from the analysis of each individual movie resulted in a list of individual particle contrasts, which were converted to mass using the corresponding C2M calibration. From these data, kernel density estimates (KDE) were generated for each sample using a Gaussian kernel with a fixed bandwidth using MATLAB (R2017b), which helped eliminate variations due to total particle counts between experiments. Bandwidth values varied between the different proteins, and were determined by experimental noise, where noisier data required larger bandwidths. Bandwidth values were 15 (Cohesin and Complex I), 20 (APC/C and NPC) and 30 (proteasome) kDa. To estimate the mole fractions of the different species, the KDEs were then fitted to a sum of several Gaussian functions. The number of Gaussians chosen as well as the respective center of mass values were identified based on the apparent number of peaks in the KDE plots. This included the presence of peak shoulders and the existence of (sub-)complexes in a given sample. We fitted the Gaussian sum using MATLAB curve fitting tools. The relative amount of each species was calculated as the area of each Gaussian (i.e., $A = \sqrt {2\pi } a\sigma$, with *A*—area, *a*—amplitude and *σ*—standard deviation of the fitted Gaussian). Only relevant species were chosen and the mole fractions were calculated by renormalizing the area from the area sum of all relevant species.

All NPC crosslinking measurements were fitted to three Gaussians at 160, 260, and 410 kDa for experiments without crosslinking, and at 160, 270, and 440 kDa for the crosslinking ones (See Supplementary Fig. 5 for a typical example). All APC/C purification step analyses were fitted to four Gaussians at 490, 640, 800, 1175 kDa corresponding to the main (sub-)complex species we observed (See Supplementary Fig. 9 for a typical example). All APC/C crosslinked samples were fitted to a single Gaussian. All Proteasome salt addition analyses were fitted to seven Gaussians at 720, 890, 1050, 1550, 1750, 2350, 2550 kDa (See Supplementary Fig. 15 for a typical example). The mass difference in the 26 S peaks (1550/1750 kDa) and 30 S peaks (2350/2550 kDa) arises from proteasome binding partners that co-purify with proteasomes in a sub-stochiometric amounts (Ecm29). For the evaluation of the relative contributions of 26 S and 26 S+Ecm29 species in Fig. 3c, we used peak information from the low salt spectra (0, 50 mM), and fixed peak position and width for the higher salt spectra, where the different species could no longer be clearly resolved. Since negative staining analysis cannot easily distinguish between the different species of 26 S and 30 S with/without co-factors bound, we summed up the respective Gaussians to represent the amount of 26 S and 30 S species in the solution that are classified together in negative-stain analysis. In all cases described above, all repeats of measurements were fitted separately, subsequently estimating the mole fraction values, followed by a calculation of the mean and standard deviation (estimated measurement error). Extracting the mole fraction from KDE generated with different bandwidths resulted in minor differences in the mole fractions values, smaller than the differences between experimental repeats, therefore mole fraction errors were calculated based on the spread repeats.

### Correction for surface-solution concentrations discrepancies

In mass photometry, proteins bind to a surface, and thereby decrease the overall concentration of the protein in solution. Young et al.^13^ showed that the main factor affecting the binding rate of different protein (sub-)complexes to the surface is their diffusion^16^, characterized by an exponential decay in time with a rate constant roughly proportional to (molecular weight)^−1/3^. As described above, every MP measurement starts following autofocus stabilization, and therefore proteins that bind within this ‘dead-time’ (~10–15 seconds) are not recorded. This is in contrast to negative staining where particles information is ‘recorded’ from the moment the droplet is placed on the surface. This discrepancy suggests that MP measurements underestimate the abundance of smaller proteins, as compared to negative staining. Since smaller proteins diffuse faster to the surface, they are likely to be more depleted during the ‘dead-time’, and as a result a relative shift towards higher mass distributions may be observed. This potential shift can be easily accounted for by applying a diffusion correction to the counted numbers of each protein species depending on their binding rate^13^. This correction is based on the comparison between the integration over the exponential decay in binding events from sample addition (at time zero, *t* = 0) until the end of the measurement time (*t* = *t*_*f*_), and the integration over the real experimental time, which starts at *t* = *t*_*0*_ and ends at *t* = *t*_*f*_:

$$N_i = M_i\frac{{1 - e^{ - k_it_f}}}{{e^{ - k_it_0} - e^{ - k_it_f}}}$$

where *N*_*i*_ is the number of particles of species *i* counted over the full integral $(t = 0 - t_f)$, $M_i$ is the experimentally measured number of particles, and $k_i$ is the binding rate constant for species *i* and is proportional to a given molecular weight (MW), $k_i \cong \alpha \cdot (MW_i)^{ - 1/3}$. Ideally, $k_i$ is quantified for each species *i* independently, then all plotted as a function of $(MW_i)^{ - 1/3}$ to verify that molecular weight diffusion is the main contribution to different binding rates, followed by extraction of the proportionality prefactor, *α*, a parameter that incorporate the physical properties related to the binding affinity to the glass as well as diffusion (which are not related to molecular weight). It is important to note here that to a first approximation *α* is insensitive to the molecular weight of the protein. Young et al. showed that for most proteins, this approximation broadly holds, and quantified the proportionality prefactor as $\alpha = 0.3\,{\mathrm{s}}^{ - 1} \cdot {\mathrm{kDa}}^{1/3}$ (calculated from the original data of the paper).

Since the different species of all APC/C and proteasome samples overlap, a quantification of $k_i$ for each species is not possible in our work. Therefore, we applied a weight-average approach to calculate *α* from the landing rate of all particles in a movie and their weighted average mass, which was very similar across all movies (*α* = 0.2 and ${\mathrm{0}}{\mathrm{.3s}}^{ - 1} \cdot {\mathrm{kDa}}^{1/3}$ for proteasome and APC/C experiments, respectively). The similarity between *α* values extracted in this work and by Young et al. supports our assumption that differences in diffusion based on differences in molecular weight is the dominant mechanism governing variation in the binding rates of different species. Due to the overlap of main species in the mass distributions, we could not exclude the possibility that other factors contribute to the binding affinity of different species to the glass, although, as discussed below, this correction produces only a minor change to the mole fractions.

The correction was applied to all APC/C purification step measurements (Supplementary Fig. 11) and proteasome salt addition experiments (Supplementary Fig. 16), and compared to the negative staining distributions. Crosslinked APC/C mole fractions were not diffusion-corrected as their KDEs exhibit only one distinct Gaussian peak. As expected, raw, uncorrected, distributions underestimated the abundance of smaller species compared to nsEM (Supplementary Fig. 11—APC/C, Supplementary Fig. 16—proteasome), while the corrected distributions exhibit excellent agreement. Supplementary Tables 2, 4 summarize the raw and corrected mole fraction values (Supplementary Table 2—APC/C, Supplementary Table 4—proteasome). It is worth mentioning that this correction is a fairly subtle detail, shifting the mole fraction values by only a small percentage (≤3%, Supplementary Tables 2 and 4).

### Crosslinking NPC-III protein

For the crosslinking of NPC-III, the protein was incubated at a concentration of 0.18 mg mL^−1^ (434 nM of complex) with 0.1% glutaraldehyde for 5 min on ice in a total volume of 9 μL, before being quenched with 1 μL of quenching buffer (crosslinking protocol was inspired from^35^). Samples taken from this final, quenched, solution were diluted 10-fold in buffer immediately before mass photometry measurements. From each reaction volume, three independent measurements were taken, with the pre-measurement dilution performed separately for each one. Three independent reactions were carried out, resulting in a total of nine measurements of the crosslinked species. For the measurements of the complex without crosslinking, the complex was diluted 10-fold from the stock concentration of 0.2 mg mL^−1^ (482 nM of complex), again immediately before measurement. This procedure was repeated for nine independent measurements of the complex without crosslinker. Crosslinking experiments were also performed as a function of incubation time. For this, the procedure described above was repeated with incubation times of 5, 10, 15, and 20 min (Supplementary Fig. 6). For each time point, two measurements were taken, and two measurements of the complex straight after dilution without crosslinking were also taken. The buffer used throughout was 10 mM HEPES, pH 7.5, 150 mM NaCl, 1 mM DTT, and 0.1 mM EDTA. The quenching buffer contained 8 mM aspartate (Asp) and 2 mM lysine (Lys).

### Mass shift due to crosslinking of NPC-III protein

There are 160 lysine residues (lys) in NPC-III. Assuming all those residues bind a glutaraldehyde molecule (100 Da), the full complex mass should increase by 16 kDa. The quenching buffer includes 0.8 mM Asp (133 Da) and 0.2 mM Lys (146 Da), which can each quench glutaraldehyde, adding another ~22 kDa per NPC-III assembly. In total, the crosslinking procedure could add up to ~38 kDa per NPC-III assembly. The observed mass shift in our experiments was 27 kDa (Fig. 1f), within the expected range.

### Negative staining

All negative staining grids were prepared with the exact protein samples and in the same concentrations used for MP measurements.

### Sample preparation, data collection, and image processing

The samples were stained with 2% (w/v) uranyl acetate. Carbon-coated grids were glow-discharged using EM ACE600 sputter coater (*Leica*) for 30 seconds at ~20 mA. Four µl of the sample was applied on the glow-discharged grid and incubated for several seconds. The excess liquid was blotted off using filter paper. The grid was washed three times with a water droplet. Four µl of 2% uranyl acetate was applied on the grid with adsorbed sample and incubated for 1 min. The excess stain was blotted off using a filter paper. The grids were air-dried before micrograph recording. Images were recorded on an FEI Tecnai G^2^ 20 (*FEI)* transmission electron microscope at a magnification of ×62k (1.85 Å pixel^−1^). Particle picking was done using CrYOLO^36^ after which the particles were transferred to cowEyes (https://www.cow-em.de/) for subsequent rounds of 2D classifications. After final 2D classification, clean 2D class averages were extracted and representative 2D classes were visualized using RELION v3.0^37^. Simulated 2D class averages where generated by converting the deposited pdb model of the APC/C into a 3D volume using EMAN2^38^. The Platform model was generated by removing relevant subunits in Chimera before converting into 3D. Relion was then used to generate random 2D projections.

### Rotary shadowing imaging—Cohesin

Cohesin trimer was first diluted to a concentration of approximately 0.1 mg mL^−1^ in 50 mM sodium phosphate buffer pH 7.6 (including 150 mM NaCl, 5% glycerol and 0.5 mM TCEP) and subsequently diluted 1:1 in spraying buffer, containing 200 mM ammonium acetate and 60% (v/v) glycerol, pH adjusted to 7.6. After dilution, the samples were sprayed onto freshly cleaved mica chips (Agar Scientific, UK) and immediately transferred into a BAL-TEC MED020 high vacuum evaporator (BAL-TEC, Liechtenstein) equipped with electron guns. While rotating, samples were coated with 0.7 nm Platinum (BALTIC, Germany) at an angle of 4-5°, followed by 7 nm Carbon (Balzers, Liechtenstein) at 90°. The obtained replicas were floated off from the mica chips, picked up on 400 mesh Cu/Pd grids (Agar Scientific, UK), and inspected in an FEI Morgagni 268D TEM (FEI, The Netherlands) operated at 80 kV. Images were acquired using an 11 megapixel Morada CCD camera (Olympus-SIS, Germany).

### Protein production, purification, and measurement condition

Respiratory complex I: Respiratory complex I was overproduced in strain BW25113 Δ*nuo*/pBAD*nuo nuoF*_*his*_ and solubilization of membrane proteins occurred by adding MNG-3 dropwise to a final concentration of 2% (w/v). Extracted proteins were loaded onto an anion-exchange Fractogel EMD TMAE Hicap column (50 mL). After washing with 149 mM NaCl, bound proteins were eluted in a linear gradient from 149 to 350 mM NaCl. Fractions with NADH/ferricyanide oxidoreductase activity were pooled and loaded onto an Probond Ni^2+^-IDA affinity column (35 mL). After washing with 140 mM imidazole, bound proteins were eluted in a single step with 260 mM imidazole. Fractions showing NADH/ferricyanide oxidoreductase activity were concentrated and applied onto a Superose 6 (24 mL) size-exclusion column. Peak fractions were concentrated to 30 µl (Amicon Ultra-15, 100 kDa MWCO) and stored in 50 mM MES/NaOH, 50 mM NaCl, 5 mM MgCl_2_ and 0.005% MNG-3 (pH 6.0) at −80 °C. MP and nsEM measurements were performed with 12.5 nM complex I after a drop dilution with its storing buffer without MNG-3. The sample was measured within 15 mins to avoid any protein aggregation due to lower MNG-3 concentration.

Cohesin (trimers): SF9 insect cells were transfected for 48 h with bacmids harboring homo sapiens (hs) SMC1, hs SMC3-FLAG and hs RAD21-Halo-His14 in the pBig1a expression vector^39^. Cells were collected by centrifugation, washed with 1xPBS and snap frozen in liquid nitrogen. Cells were lysed by douncing 25 times in Lysis Buffer (50 mM NaPO4, pH 7.6, 500 mM NaCl, 5% glycerol, 0.05% Tween, 10 mM Imidazole) supplemented with EDTA-free protease inhibitor cocktail (Roche), 1 mM phenyl-methyl-sulfonyl-fluoride (PMSF), 1 mM benzamidine, and 3 mM beta-mercapto-ethanol (bME). Insoluble material was removed by centrifugation (18.5 krpm, LYNX, 35 min, 4 °C) and the supernatant applied for 3 h at 4 °C to 5 mL of Toyopearl AF-Chelate-650M resin (Tosoh) precharged with Ni^2+^-ions. Beads were collected by low speed centrifugation, washed in batch two times with 10 column volumes (CVs) of Lysis buffer supplemented with 5 mM imidazole, collected in a glass column in 5 CVs of Lysis buffer plus 5 mM imidazole and eluted with 5 CVs elution buffer (50 mM NaPO_4_, pH 7.6, 150 mM NaCl, 5% glycerol, 300 mM Imidazole). The eluate was incubated for 3 h at 4 °C with 5 mL of FLAG M2 Agarose beads (Sigma), collected in a glass column, washed with 2 × 5 CVs of FLAG-Buffer (50 mM NaPO_4_, pH 7.6, 100 mM NaCl, 5% glycerol, 50 mM Imidazole) and eluted with 5 × 1 CV of FLAG-buffer supplemented with 0.25 mg mL^−1^ 3xFLAG peptide. The eluate was bound to 75 µL of POROS HS resin (Thermo) for 30 min at 4 °C. The beads were collected in a disposable plastic column, and bound cohesin eluted with 3 × 150 µL High Salt Buffer (50 mM NaPO_4_, pH 7.6, 750 mM NaCl, 5% glycerol, 50 mM Imidazole, 0.5 mM TCEP). The eluate was dialyzed overnight against Dialysis Buffer (50 mM NaPO_4_, pH 7.6, 100 mM NaCl, 5% glycerol, 0.5 mM TCEP), aliquoted, snap-frozen in liquid nitrogen and stored at −80 °C. Protein concentrations were determined by the Bradford Assay, assuming a molecular weight of 397 kDa. MP measurements of Cohesin trimers were performed at 21 nM, diluted from stock solution using its storing buffer.

### Nuclear pore complex (NPC)

NPC—Construct generation: NPC-I: Nup82_1-854_, Nup159_1072-1447_, and Nsp1_507-718_ were cloned from GeneArt Strings (ThermoFisher Scientific) into modified pET-Duet plasmids containing three expression cassettes. The first cassette contained Nup159_1072-1447_ fused to an N-terminal TEV protease cleavable His_6_-Avi-MBP tag. Nup82_1-854_ and Nsp1_507-718_ were cloned into the second and third cassette, respectively, without any modification. Nup145N_868-1004_ was cloned from *M. thermophila* into a pET plasmid introducing an N-terminally fused human rhinovirus 3 C (3 C)–cleavable His_10_-Arg_8_-SUMO tag. The tetrameric complex, Nup82-Nup159cc-Nsp1cc-Nup145N_APD_ is referred to as NPC-I.

NPC-II: Nup120_952–1241_, Nup145C_1005–1791_, Nup85_257–1181_, and full-length Sec13 were cloned from *M. thermophila*. To increase stability, Sec13 was spliced between Nup145C_1005-1232_ and Nup145C_1233–1791_ to generate a structure-based fusion protein. The Nup145C_1005-1232_-Sec13-Nup145C_1233–1791_ protein construct was N-terminally tethered to a 3C–cleavable SUMO tag. Nup85_257–1181_ was N-terminally fused with a 3C–cleavable His_10_-Arg_8_-SUMO. Nup120_952–1241_ was C-terminally fused with a His_10_ tag. All constructs were cloned into pET-derived plasmids. The tetrameric complex Nup120- Nup145C-Sec13-Nup85 is referred to as NPC-II for simplicity.

NPC—Protein production and purification: *Escherichia coli* LOBSTR-RIL(DE3) (Kerafast)^40^ cells were co-transformed with vectors, and protein production was induced with 0.2 mM IPTG at 18 °C for 12–14 h.

Production of NPC-III: For simplicity, the octameric Nup82-Nup159cc-Nsp1cc-Nup145N_APD_-Nup120-Nup145C-Sec13-Nup85 is referred as NPC-III and was produced as follows. NPC-II was purified as in ref. ^41^. Cells expressing trimeric Nup82_1-854_-Nsp1_507-718_-Nup159_1072-1447_ were collected by centrifugation at 6,000 *g*, resuspended in lysis buffer (50 mM potassium phosphate, pH 8.0, 500 mM NaCl, 3 mM β-mercaptoethanol (βME) and 1 mM PMSF) and lysed with an LM20 microfluidizer (Microfluidics). The lysate was cleared by centrifugation at 12,500 × *g* for 25 min. The soluble fraction was incubated with amylose resin (NEB) for 30 min at 4 °C. After washing of the beads with lysis buffer, the protein was eluted (10 mM maltose, pH 8.0, 150 mM NaCl, and 3 mM βME). The Nup82_1-854_-Nsp1_507-718_-Nup159_1072-1447_ amylose eluate was incubated with TEV and dialyzed overnight at 4 °C (10 mM Tris-HCl, pH 8.0, 150 mM NaCl, 0.1 mM EDTA and 1 mM DTT). Purified Nup145N_APD_ was incubated with trimeric Nup82_1-854_-Nsp1_507-718_-Nup159_1072-1447_ and the assembled tetrameric NPC-I complex separated by size-exclusion chromatography on Superdex S200 (GE Healthcare) in 20 mM HEPES-KOH, pH 7.4, 0.1 mM EDTA and 1 mM DTT. NPC-I and NPC-II were mixed and NPC-III isolated by size-exclusion chromatography on Superdex S200 (20 mM HEPES-KOH, pH 7.4, 0.1 mM EDTA and 1 mM DTT).

### APC/C purification and sample preparation

Recombinant APC/C was expressed in High Five insect cells (Thermo Scientific) as described in^27,39^. Briefly, APC/C was expressed with a Twin-Strep(II)-tag on the C-terminus of APC4 and purified using Strep-Tactin Sepharose (IBA Life Sciences) affinity chromatography followed by ion-exchange chromatography and SEC. Final buffer conditions were 20 mM Hepes pH 8, 200 mM NaCl, 1 mM DTT. Crosslinked APC/C EM samples were prepared as described in ref. ^27,28^.

All APC/C samples were diluted to working concentration using APC/C buffer (20 mM Hepes pH 8, 200 mM NaCl), which varied between samples to optimize background noise and particles counts. The concentrations were Strep (3, 4, 5) 12 nM, IEX (26, 32, 38, 48) 5 nM, SEC (9, 10, 12, 13) 15 nM. APC/C^CDH1^-UBE2C, and APC/C^CDH1^-UBE2S traps were measured directly after buffer exchange using a Zeba spin column (Pierce) to remove glycerol.

### Proteasome

Proteasome complexes were purified from bovine heart extract by the protocol adapted from ref. ^32^. Briefly, bovine heart was homogenized in purification buffer (25 mM BisTris, pH 6.5, 50 mM KCl, 5 mM MgCl_2_, 10% glycerol, 4 mM ATP and 1 mM DTT) supplemented with 0.1% Triton and 0.1 mM PMSF and cleared in an Optima XE-90 Ultracentrifuge, Ti45 rotor (Beckman Coulter) for 1 h at 100,000 × *g* at 4 °C. The final extract was prepared by two-step protein precipitation with 4 and 20% PolyEthyleneGlycol8000 (PEG8000). Precipitated proteins were dissolved in purification buffer. Proteasomes were affinity purified with the bait protein GST-Ubl and magnetic beads (MagneGST™ Glutathione Particles, Promega) and eluted with purification buffer containing 25 mM reduced L-glutathione. Proteasome samples were concentrated and applied on 10–30% sucrose gradients (purification buffer containing 10 or 30% (w/v) sucrose). Gradients were run in Optima XE-90 Ultracentrifuge, SW60Ti rotor (Beckman Coulter) for 16 h at 100,000 × *g* and 4 °C. Gradients were manually fractionated into 200 µL fractions and protein concentrations were determined by the Bradford assay (Protein Assay Dye Reagent Concentrate 5×, Bio-Rad).

### Sample preparation under different conditions

Prior to mass photometry measurements, proteasome samples (1 µM) were buffer exchanged (Zeba Micro Spin Desalting Columns, Thermo Fisher Scientific) into 25 mM BisTris, 50 mM KCl, 5 mM MgCl_2_, 20% glycerol, 4 mM ATP and 1 mM DTT and diluted 2×. For measuring proteasome stability in the presence of NaCl, the proteasome sample was first diluted to 50 nM and then NaCl was added to a final concentration of 50, 100, 250, and 500 mM, and samples were incubated on ice for 2 h. For measuring proteasome stability under different nucleotide conditions either apyrase (100 mU), which hydrolyzes ATP and ADP to AMP, hexokinase (100 mU) and D-glucose (20 mM), which hydrolyses ATP to ADP in a reaction that results in generation of glucose-6-phosphate, apyrase (100 mU) and proteasome inhibitor epoxomicin (50 µM), which stabilized proteasome complex were added to 2x diluted proteasomes and incubated for 2 h at 37 °C. Before measurements, proteasome samples were diluted to 50 nM. We could not detect either apyrase or hexokinase at the detection conditions used, which were optimized to suppress buffer background and therefore limited detection to >300 kDa species.

### Reporting summary

Further information on research design is available in the Nature Research Reporting Summary linked to this article.

## Supplementary information

Supplementary Information

Peer Review

Reporting Summary

Description of Additional Supplementary Files

Supplementary Movie 1

## Source Data

Source Data

## Data Availability

All data necessary to support the conclusions are available in the manuscript or supplementary materials, and available from the corresponding authors upon reasonable request. All mass photometry data are deposited in the University of Oxford Research Archive (DOI, 10.5287/bodleian:xQgR57q59). All EM data are available upon request to the lead authors due to its large data size. The source data underlying Figs. 1b,d,f,g, 2c-e, 3b-e, and Supplementary Figs. 2-9, 11, 14-19 are provided as a Source Data file.

## Code availability

AcquireMP and DiscoverMP are available from Refeyn Ltd.
