# Supplementary material for: Quantifying the heterogeneity of macromolecular machines by mass photometry
Source: Nat Commun. 2020 Apr 14;11:1772. doi: 10.1038/s41467-020-15642-w (PMC7156492; doi:10.1038/s41467-020-15642-w)
Supplement: Supplementary file 1 — Supplementary Information [file 41467_2020_15642_MOESM1_ESM.pdf]

Supplementary information for

Quantifying the heterogeneity of macromolecular machines by mass photometry

Sonn-Segev et al.

Supplementary Movie 1: First 5 seconds (real time) of a representative ratiometric movie of Complex I at 12.5 nM binding non-specifically to a glass coverslip. The field of view is $2.9 \times 10.8\ \mu m^2$ and the raw frames were saved at 200 Hz, while the sliding ratiometric processing was applied with a frame summing of 5 frames. The movie is played back at 10 Hz.

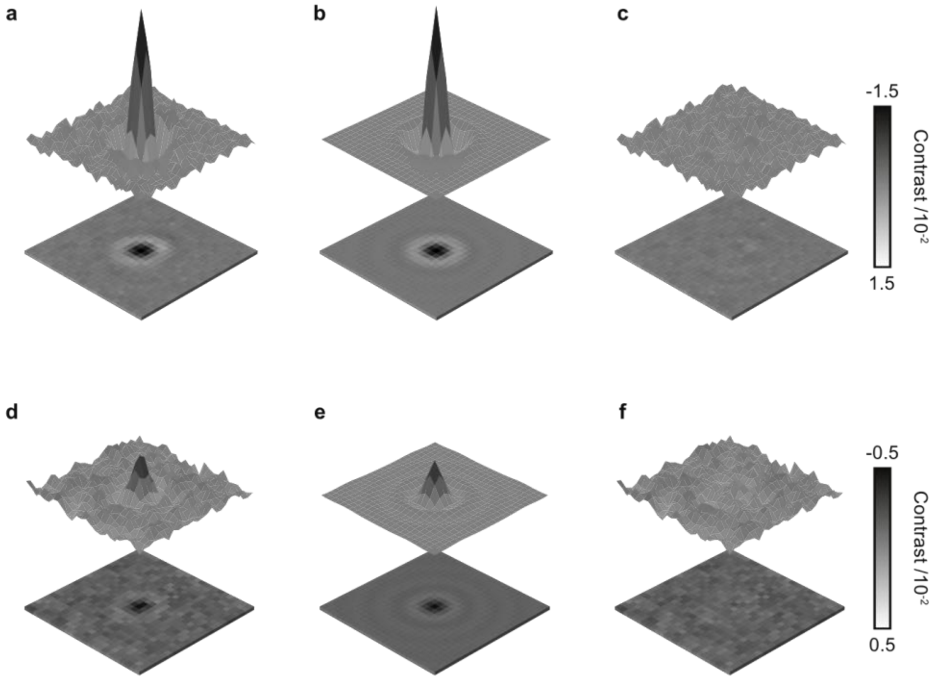

Supplementary Figure 1: Point spread function (PSF) fitting for mass photometry. Experimental (**a**, **d**) and fitted (**b**, **e**) PSF and the corresponding residual (**c**, **f**) for two particles with large (**a**, **b**, **c**) and small (**d**, **e**, **f**) signal-to-noise (SNR) ratios, SNR=12 and 3.5, respectively.

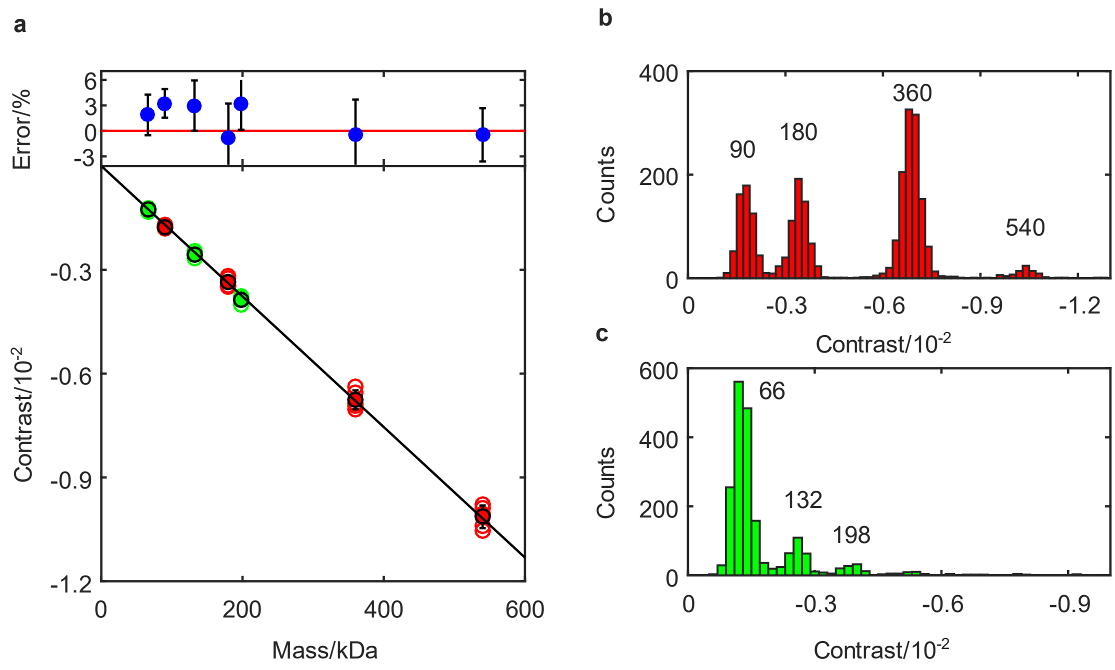

Supplementary Figure 2: Contrast-to-mass (C2M) calibration curve. **a**, The contrasts of two proteins of known mass with different oligomeric states (Protein 1: 90, 180, 360, 540 kDa - red; Protein 2: 66, 132 and 198 kDa – green) are plotted vs their known mass. The black line is the fit to the data according to $y = bx$, with b representing the C2M calibration factor. The fitting residuals are presented in the above panel as a percentage mass error. The contrast distributions of Protein 1 (**b**) and Protein 2 (**c**) exhibit the relative abundances of the different oligomers. The numbers above each correspond to their mass in kDa. In principle, any mixture or combination of proteins with known mass can be used for mass calibration. We chose oligomeric proteins because they provide multiple reference points in a single measurement, thereby accelerating and simplifying the process. Error bars are calculated as standard deviation of contrast (or residue) from different repeats. Source data are provided as a Source Data file.

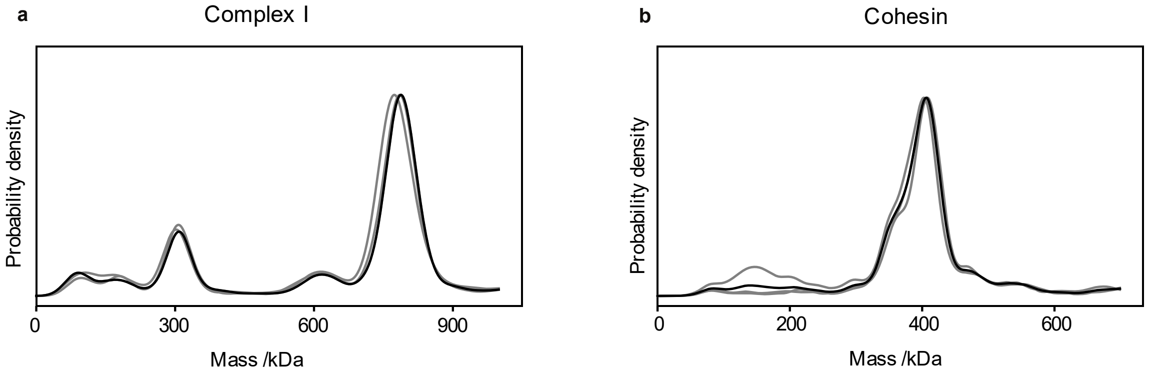

Supplementary Figure 3: Kernel density estimates (KDE) for repeats of mass photometry measurements for Complex I (**a**) and Cohesin (**b**) showing the reproducibility of MP for those complexes. Grey lines correspond to probability densities of 3 different independent measurements and black line is the combined probability density calculated from all particles. Number of particles of each repeat different are $N_{\mathrm{complex\,I}} = 2024, 2177, 1801$ and $N_{\mathrm{cohesin}} = 2139, 1949, 1953$. Source data are provided as a Source Data file.

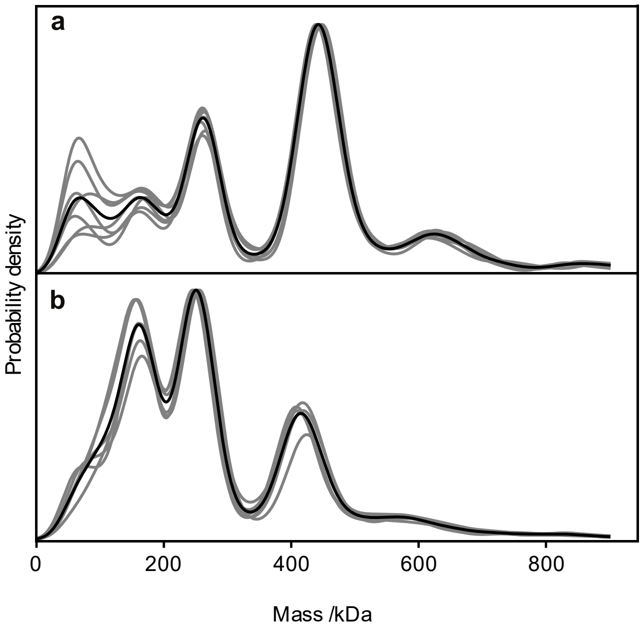

Supplementary Figure 4: Reproducibility of MP measurements for Nuclear Pore complex (NPC) before and after cross-linking. Probability density plots of MP for cross-linked NPC (**a**) for 5 min and without cross-linking treatment (**b**). Grey lines depict the probability density of 7 different independent measurements and the black line is the combined probability density. The observed increased variability at low compared to high mass is a consequence of non-unity detection efficiency for the detection parameters used. Source data are provided as a Source Data file.

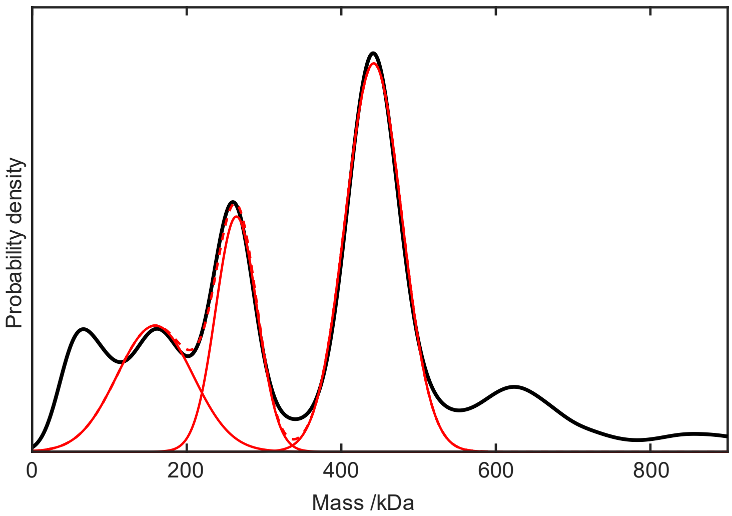

Supplementary Figure 5: Multiple Gaussian fitting to MP distributions of NPC. Black line corresponds to the experimental KDE, and red solid (dash) lines correspond to the 3 individual (sum of) fitted Gaussians. Source data are provided as a Source Data file.

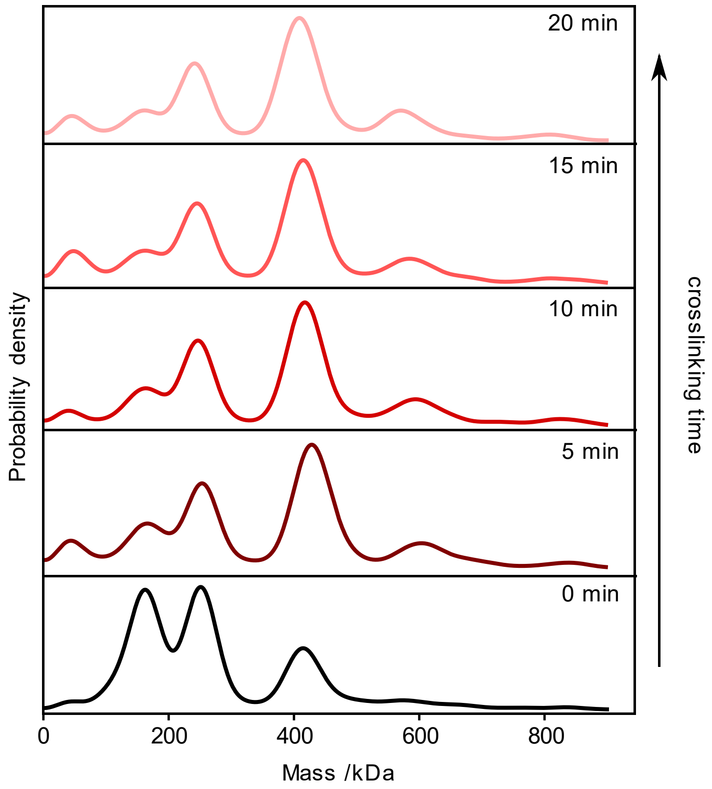

Supplementary Figure 6: Time dependent cross-linking of Nuclear Pore complex (NPC). MP probability density plots of NPC without any treatment (0 min), and after treatment with 0.1% glutaraldehyde for 5, 10, 15, 20 min, followed by quenching. Source data are provided as a Source Data file.

Supplementary Table 1. Molecular weights of individual APC/C subunits and APC/C subcomplexes. Subunits that form dimers are indicated with an asterisk. The main subcomplexes are APC/C$^{\text{Platform-APC8}}$ (523 kDa), APC/C^{Platform} (661 kDa), APC/C$^{\text{Platform+APC6}}$ (825 kDa) and the full complex APC/C (1175.5 kDa).

Subunit	Molecular weight (kDa)	Subunit	Molecular weight (kDa)	
APC1	216	APC8*	69	
APC2	94	APC11	10	
APC4	96	APC13	9	"Platform" subunits
APC5	85	APC15	15	
APC3*	92	APC10	21	
APC6*	72	APC12*	10	"Arc lamp" subunits
APC7*	67	APC16	12	

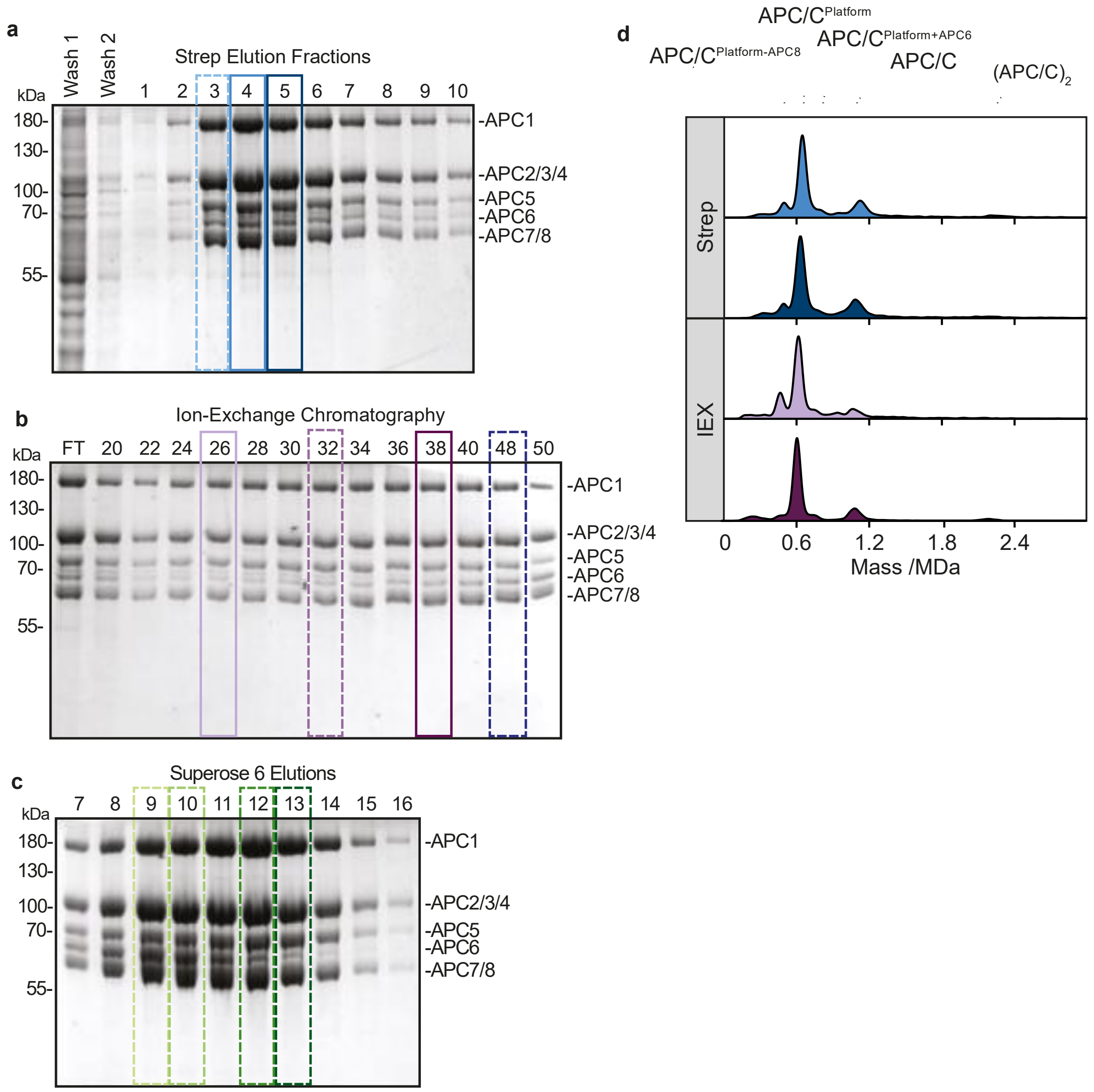

Supplementary Figure 7. SDS PAGE and MP analysis of APC/C. **a-c,** SDS PAGE analysis of APC/C purification steps. Boxed lanes indicate samples measured by MP as depicted in **d**. Dashed boxes indicate samples described in **Figure 2c-f.** Fractions 1-10 eluted from Strep-tactin (Strep) were combined for subsequent ion-exchange chromatography (IEX). Fractions 32-50 were combined for subsequent size-exclusion chromatography (SEC). **d,** MP measurements of fractions indicated in **a,b**. Source data are provided as a Source Data file.

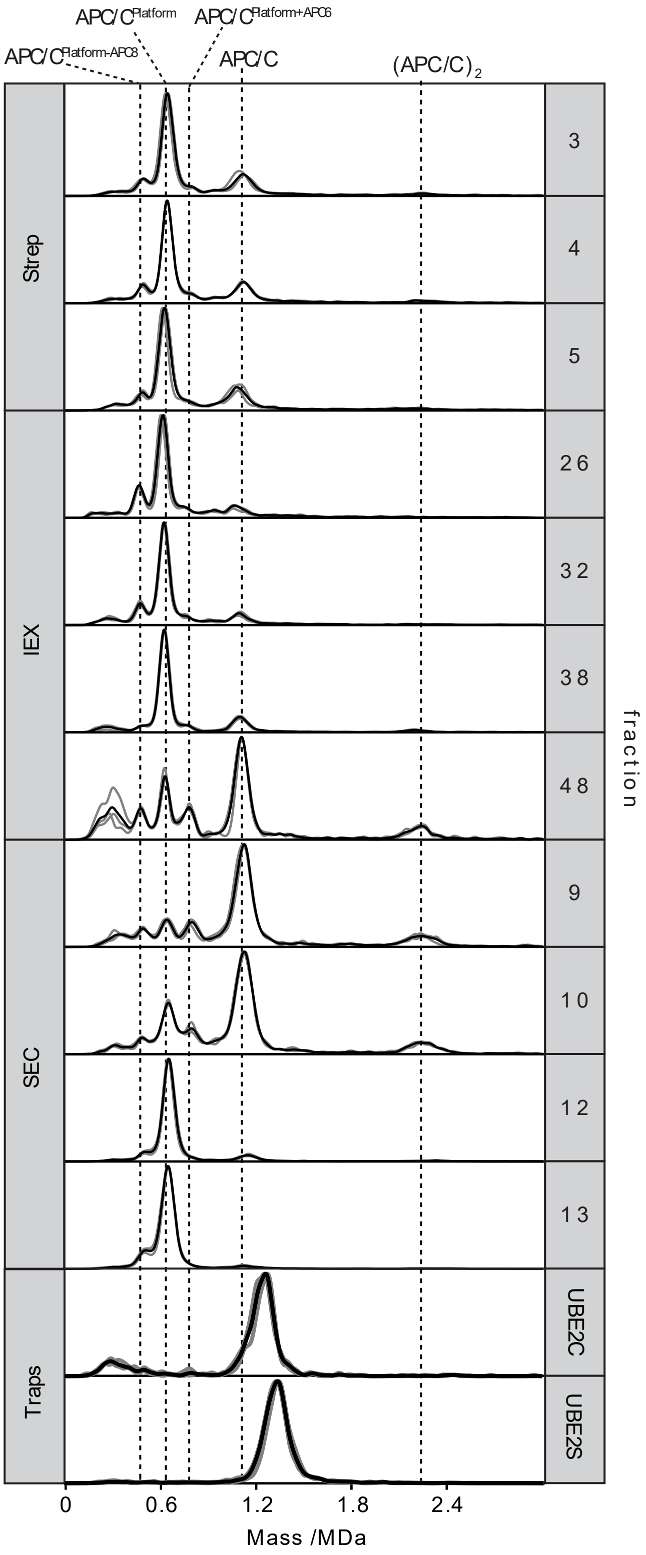

Supplementary Figure 8: Reproducibility of MP measurements for APC/C at its different purification steps, and two purified and cross-linked samples. Probability density plots of MP for different fractions of the three purification steps (Strep, IEX and SEC), and the two cross-linked APC/C: APC/C^{CDH1}-UBE2C and APC/C^{CDH1}-UBE2S traps indicated as UBE2C and UBE2S, respectively. Grey lines depict probability density of 3 different independent measurements and the black line represents the averaged probability density. Source data are provided as a Source Data file.

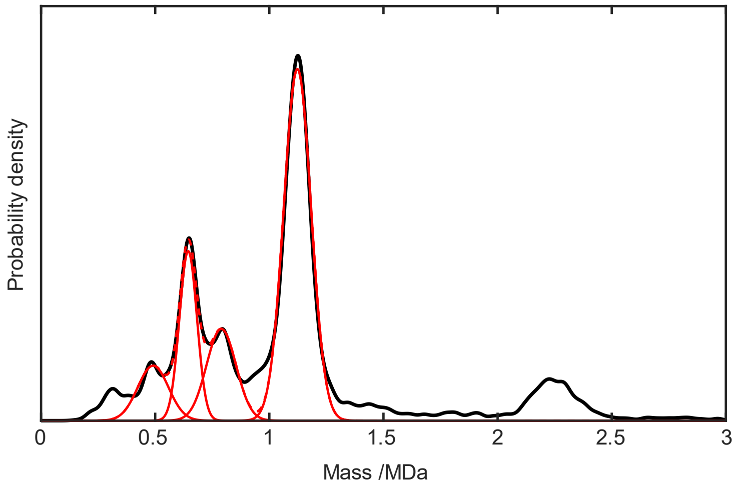

Supplementary Figure 9: Gaussian fitting to MP plots of APC/C. Four Gaussian fit to the KDE of SEC-fraction 10. The black line is the experimental KDE, and the red solid lines 4 individual fitted Gaussians, with the dashed lines representing their sum. The KDE bandwidth (20 kDa) was chosen as a compromise between the known mass resolution of the system and avoiding obvious broadening of the mass distribution as a consequence of applying the KDE. We limited the fit to the four main spectral features to avoid overfitting. Source data are provided as a Source Data file.

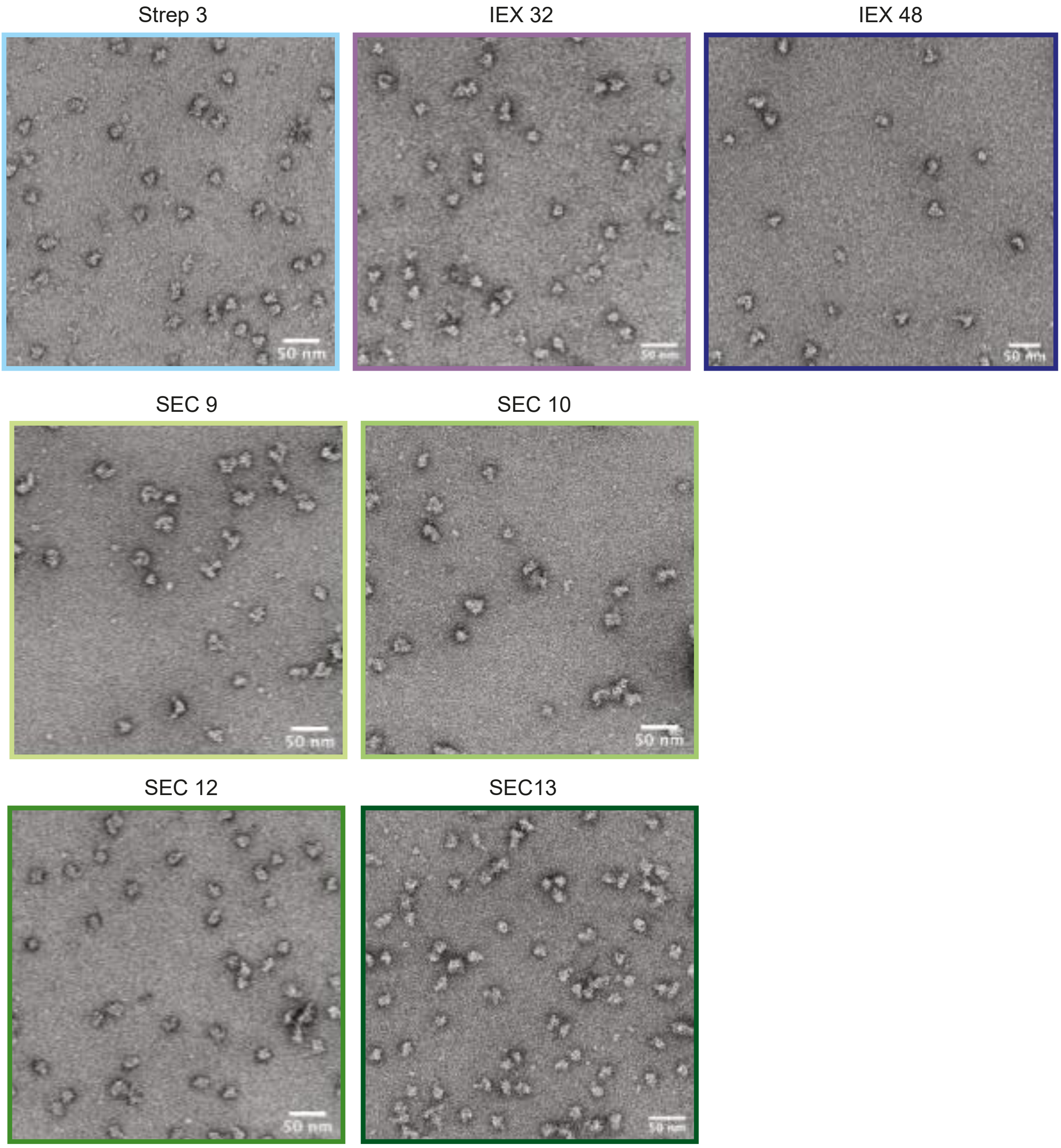

Supplementary Figure 10: Representative negative stain electron micrographs of APC/C fractions from Strep-tactin (Strep), ion-exchange chromatography (IEX), and size-exclusion chromatography (SEC) purification steps corresponding to 2D classifications depicted in **Figure 2f**. Scale bar: 50 nm.

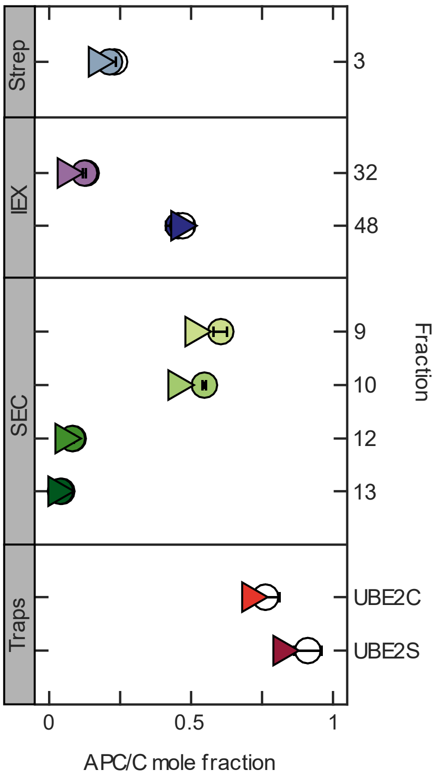

Supplementary Figure 11: Raw and diffusion-corrected mole fraction of APC/C obtained by MP, compared to nsEM. Mole fraction of full APC/C at the different steps of the purification steps (Strep, IEX and SEC) as well as for the two APC/C traps (APC/C cross-linked with cofactors) measured by MP (circles) and nsEM (triangles). ‘Raw’ MP mole fraction (empty circles) were corrected to account for the ‘dead-time’ (filled circles), resulting in a decrease in full APC/C fraction. Error bars are calculated as standard deviation of mole fractions from different repeats. Source data are provided as a Source Data file.

	Strep 3	Strep 4	Strep 5	IEX 26	IEX 32	IEX 38	IEX 48	SEC 9	SEC 10	SEC 12	SEC 13	Traps UBE2C	Traps UBE2S
MP	0.23	0.22	0.24	0.13	0.13	0.17	0.47	0.63	0.56	0.084	0.044	0.75	0.89
MP corrected	0.21	0.20	0.22	0.13	0.12	0.16	0.45	0.60	0.54	0.078	0.040	-	-
nsEM	0.17				0.06		0.46	0.51	0.45	0.052	0.027	0.71	0.82

Supplementary Table 2: APC/C Mole fraction at different steps of purification quantified by MP and nsEM. MP mole fractions were corrected for diffusion.

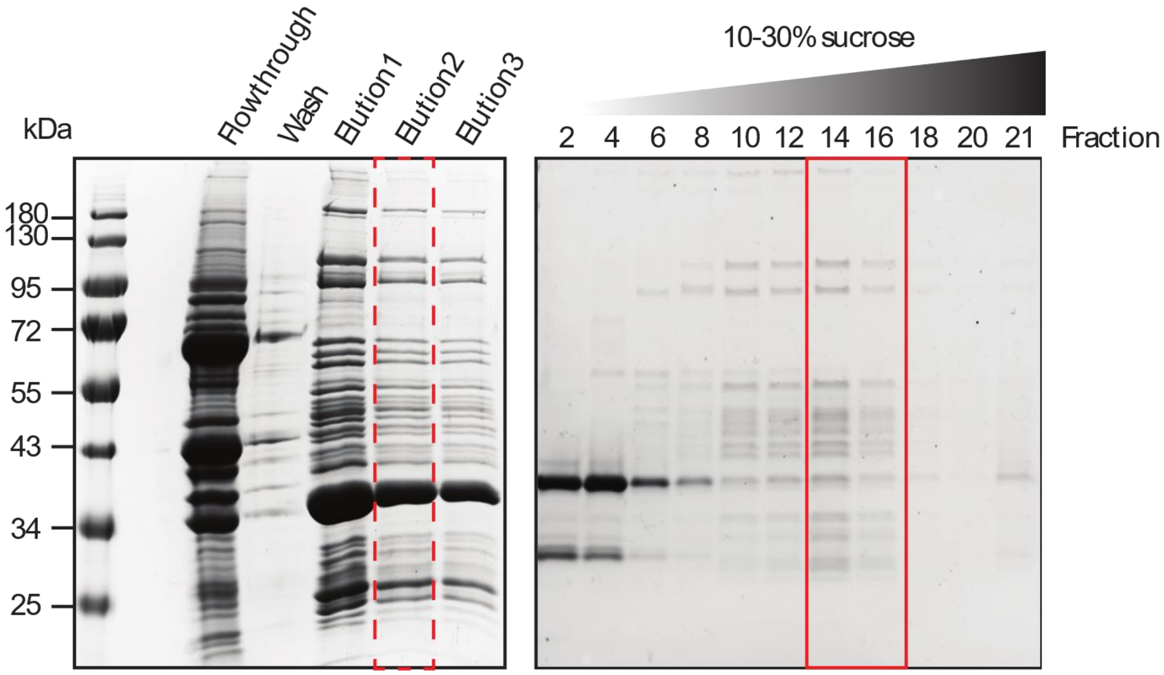

Supplementary Figure 12. SDS PAGE analysis of affinity purification of proteasome complexes from bovine heart. Elution2 is shown in **Fig. 3a**. Elution samples were applied on a 10-30% sucrose gradient. The boxed gradient fractions (14, 15 and 16) were pooled and used for MP and nsEM measurements.

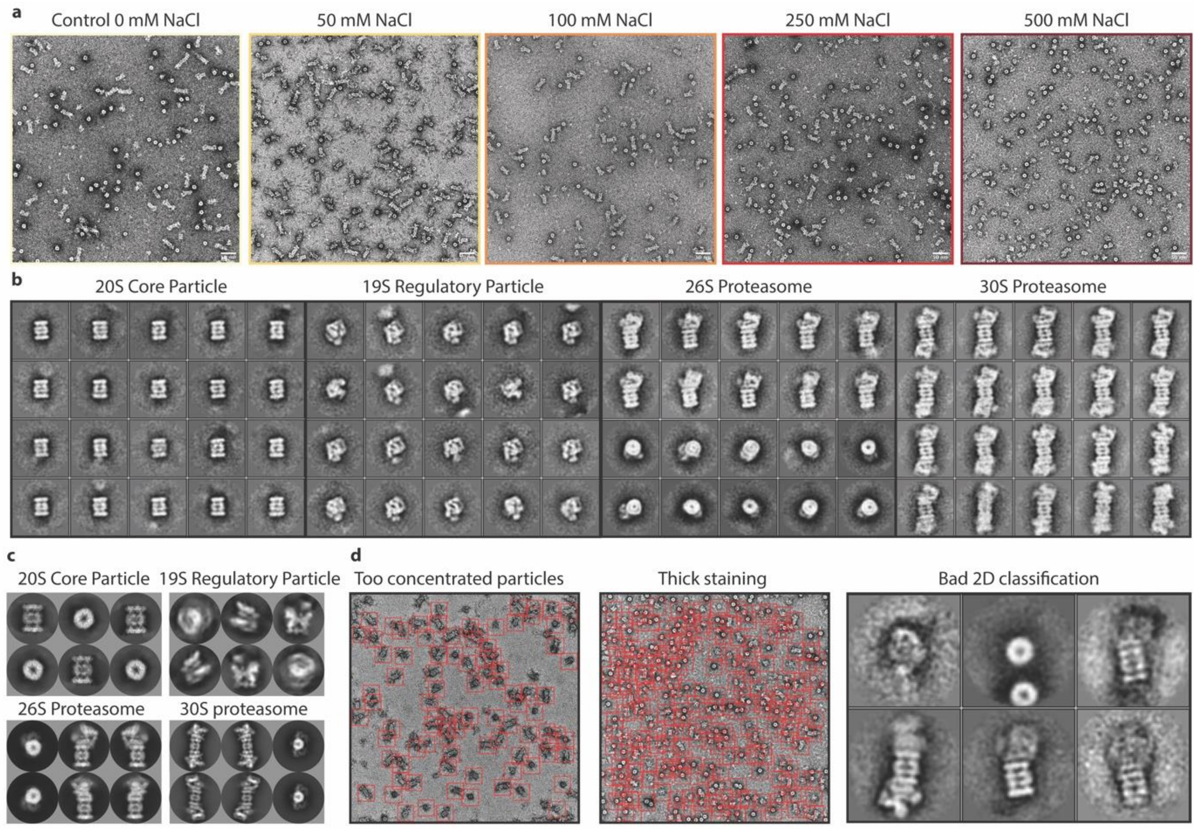

Supplementary Figure 13: Negative stain transmission electron microscopy analysis of proteasome samples. **a,** Representative negative stain electron micrographs for each salt concentration. Scale bar: 50 nm. **b,** Representative experimental 2D class averages of all proteasome complexes and subcomplexes. **c,** 2D projections (sampling at 120°) showing different views of proteasome complexes and subcomplexes. **d,** Examples of pitfalls in negative stain transmission electron microscopy sample preparation and data processing.

Supplementary Table 3: Molecular weights of individual proteasomal subunits. 20S core particle (20S CP: 760 kDa) and 19S regulatory particle (19S RP: 893 kDa) are the subcomplexes of 26S (20S CP–19S RP: 1653 kDa) and 30S (19S RP–20S CP–19S RP: 2546 kDa).

20S core particle subunits	Molecular weight (kDa)	19S regulator particle subunits	Molecular weight (kDa)
Proteasome subunit alpha-type 1	29.57	26S proteasome regulatory subunit 7 (Rpt1)	48.60
Proteasome subunit alpha-type 2	25.88	26S proteasome regulatory subunit 4 (Rpt2)	49.185
Proteasome subunit alpha-type 3	28.39	26S proteasome regulatory subunit 6B (Rpt3)	47.34
Proteasome subunit alpha-type 4	29.47	26S proteasome regulatory subunit 10 (Rpt4)	44.05
Proteasome subunit alpha-type 5	26.39	26S proteasome regulatory subunit 6A (Rpt5)	49.204
Proteasome subunit alpha-type 6	27.38	26S proteasome regulatory subunit 8 (Rpt6)	45.6
Proteasome subunit alpha-type 7	27.85	26S proteasome non-ATPase regulatory subunit 2 (Rpn1)	100.19
Proteasome subunit beta-type 1	26.23	26S proteasome non-ATPase regulatory subunit 1 (Rpn2)	105.836
Proteasome subunit beta-type 2	22.88	26S proteasome non-ATPase regulatory subunit 3 (Rpn3)	60.92
Proteasome subunit beta-type 3	22.98	26S proteasome non-ATPase regulatory subunit 12 (Rpn5)	60.92
Proteasome subunit beta-type 4	29.01	26S proteasome non-ATPase regulatory subunit 11 (Rpn6)	53.02
Proteasome subunit beta-type 5	28.59	26S proteasome non-ATPase regulatory subunit 6 (Rpn7)	47.43
Proteasome subunit beta-type 6	25.53	26S proteasome non-ATPase regulatory subunit 7 (Rpn8)	45.5
Proteasome subunit beta-type 7	30.009	26S proteasome non-ATPase regulatory subunit 13 (Rpn9)	36.73
-	-	26S proteasome non-ATPase regulatory subunit 4 (Rpn10)	42.84
-	-	26S proteasome non-ATPase regulatory subunit 14 (Rpn11)	41.36
-	-	26S proteasome non-ATPase regulatory subunit 8 (Rpn12)	34.57
-	-	26S proteasome non-ATPase regulatory subunit 15 (Sem1)	32.52

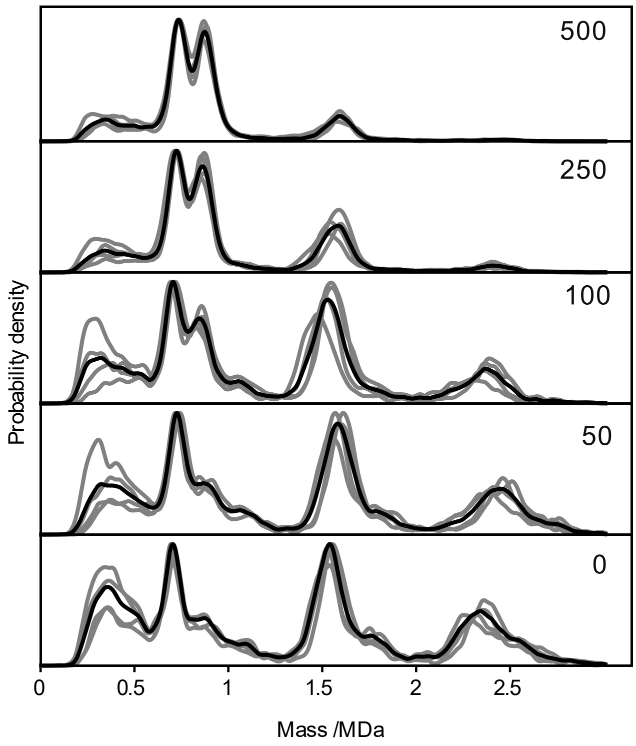

Supplementary Figure 14: Reproducibility of MP measurements for proteasome at different NaCl concentrations. Probability density plots of MP for different NaCl concentrations in mM. Grey lines correspond to the probability density of 4 different independent measurements (and at two different days) and the black line is the combined probability density. Source data are provided as a Source Data file.

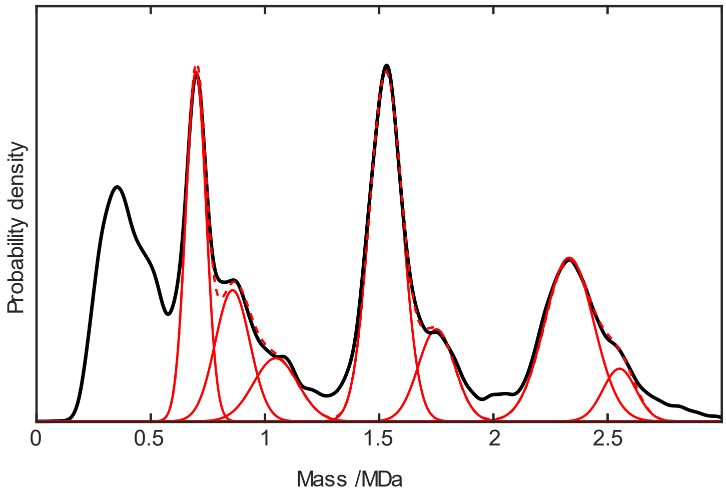

Supplementary Figure 15: Gaussian fitting to MP distribution for the proteasome. Seven Gaussian fitting to the KDE at 0 mM NaCl. Black line corresponds to the experimental KDE, and red solid (dash) lines correspond to the 7 individual (sum of) fitted Gaussians. KDE bandwidths and number of fitting parameters was chosen as discussed in **Supplementary Figure 9**. Source data are provided as a Source Data file.

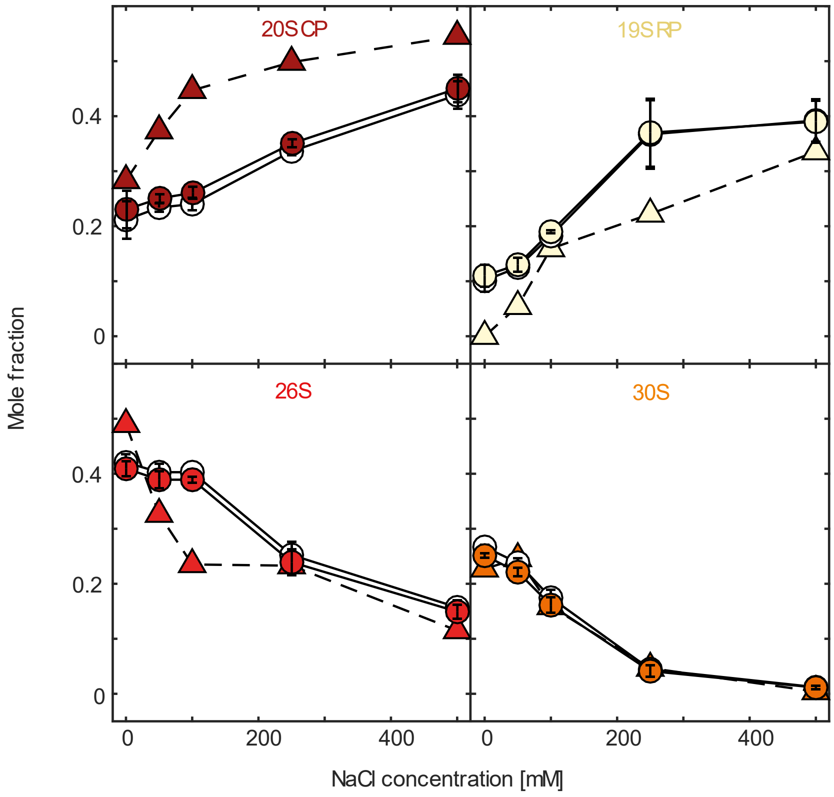

Supplementary Figure 16: Diffusion corrected mole fraction for proteasome treatment with salt. Mole fraction vs NaCl concentration for the 4 different proteasome (sub-) complexes, 20S, 19S RP, 26S and 30S, measured by MP (circles) and nsEM (triangles). 'Raw' MP mole fraction (empty circles) were corrected to account for the 'dead-time' (filled circles), resulting in an increase of the mole fractions for small species. Error bars are calculated as standard deviation of mole fractions from different repeats. Source data are provided as a Source Data file.

NaCl /mM	20S MP	20S MP corrected	20S nsEM	19S MP	19S MP corrected	19S nsEM	26S MP	26S MP corrected	26S nsEM	30S MP	30S MP corrected	30S nsEM
0	0.21	0.23	0.28	0.10	0.11	0	0.42	0.41	0.49	0.27	0.25	0.23
50	0.24	0.25	0.37	0.12	0.13	0.054	0.40	0.39	0.33	0.24	0.22	0.25
100	0.24	0.26	0.44	0.18	0.19	0.16	0.40	0.39	0.23	0.17	0.16	0.16
250	0.34	0.35	0.50	0.37	0.37	0.22	0.25	0.24	0.23	0.045	0.041	0.046
500	0.44	0.45	0.54	0.39	0.39	0.34	0.16	0.15	0.11	0.012	0.011	0.004

Supplementary Table 4: Proteasome mole fraction analysis for its different (sub-) complexes, at increasing salt concentration, quantify by MP and nsEM. MP mole fractions were corrected for the different diffusion of (sub-) complex, and are in good agreement with nsEM ones.

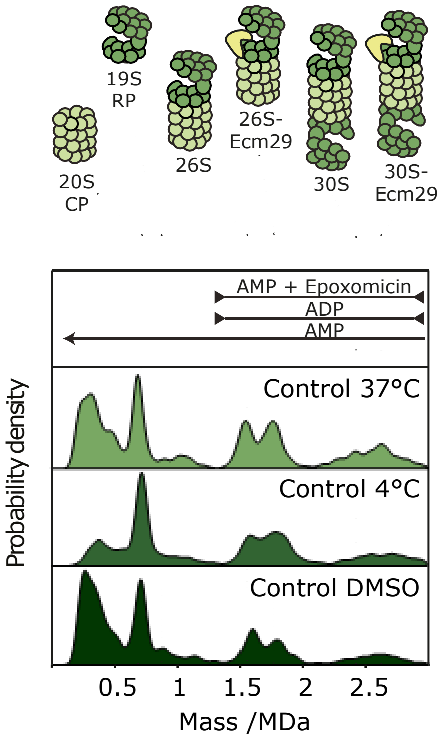

Supplementary Figure 17: MP control experiments for proteasome samples at 4°C, 37°C and in the presence of DMSO, for the proteasome sample used for different nucleotide conditions (**Fig. 3e** of main text). All measurements were performed at 50 nM. Source data are provided as a Source Data file.

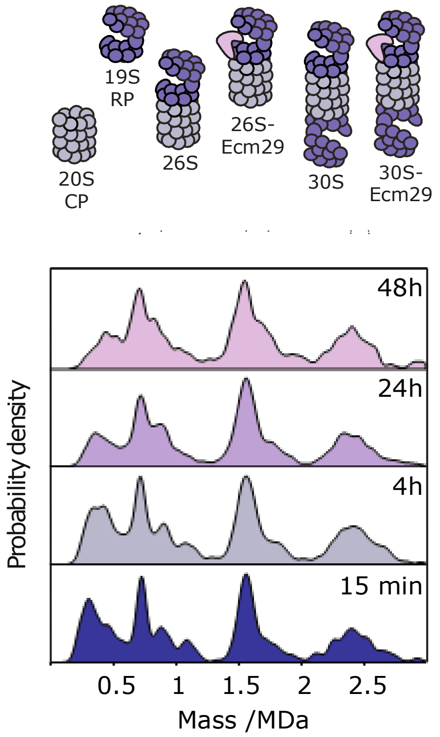

Supplementary Figure 18: MP measurements of proteasome stability. Probability density of proteasome at different time points (15 min, 4h, 24h and 48h) show that proteasome (sub)complexes are stable even after 48 hours. All samples were kept at 4°C and measured at 50 nM. Source data are provided as a Source Data file.

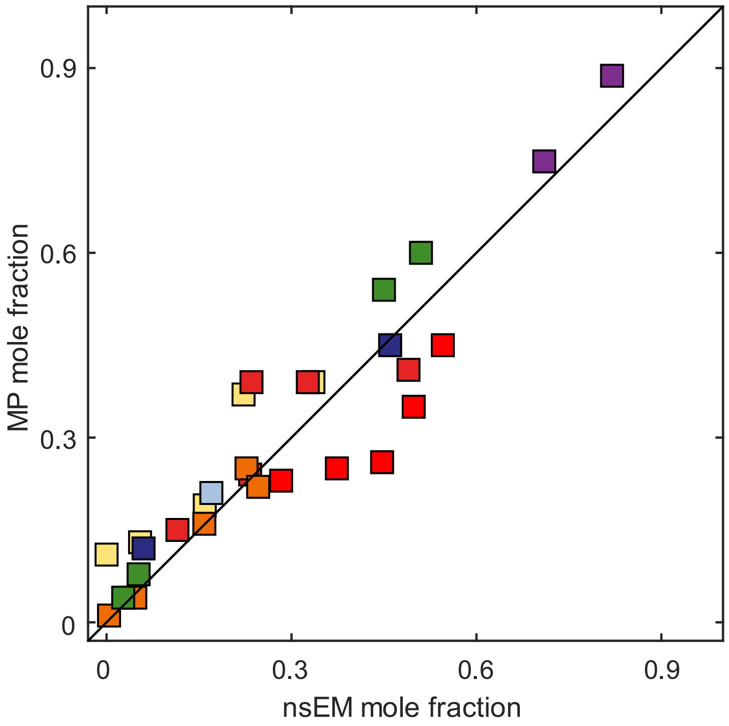

Supplementary Figure 19: Mass photometry and negative staining comparison. Scatter plot of mole fraction in MP vs nsEM for all analyzed samples of APC/C purification steps and Proteasome salt addition. Colors correspond to different protein samples: APC/C in purification steps: strep (light-blue), IEX (dark-blue), SEC (green), APC/C cross-linked (purple); Proteasome – 20S (brown), 19S (yellow), 26S (red) and 30S (orange). Solid line corresponds to $y = x$, with R^2=0.9. Source data are provided as a Source Data file.
