# Supplementary material for: Quantifying the heterogeneity of macromolecular machines by mass photometry
Source: Nat Commun. 2020 Apr 14;11:1772. doi: 10.1038/s41467-020-15642-w (PMC7156492; doi:10.1038/s41467-020-15642-w)
Supplement: Supplementary file 4 — Description of Additional Supplementary Files [file 41467_2020_15642_MOESM4_ESM.docx]

**Description of Additional Supplementary Files**

**File name: Supplementary Movie 1**
Description: First 5 seconds (real time) of a representative ratiometric movie of Complex I at 12.5 nM binding non-specifically to a glass coverslip. The field of view is 2.9×10.8 μm2 and the raw frames were saved at 200 Hz, while the sliding ratiometric processing was applied with a frame summing of 5 frames. The movie is played back at 10 Hz.
