# Supplementary material for: Quantifying the heterogeneity of macromolecular machines by mass photometry
Source: Nat Commun. 2020 Apr 14;11:1772. doi: 10.1038/s41467-020-15642-w (PMC7156492; doi:10.1038/s41467-020-15642-w)
Supplement: Supplementary file 2 — Peer Review [file 41467_2020_15642_MOESM2_ESM.pdf]

Reviewers' comments:

Reviewer #1 (Remarks to the Author):

In this manuscript, Sonn-Segev et al. show how the recent label-free method, termed mass photometry (MP), provides information on sample heterogeneity largely equivalent to that obtained by negative stain electron microscopy (nsEM). Since MP data can be collected faster than nsEM, this approach could be of interest to researchers in the field of cryo-electron microscopy and x-ray crystallography.

Overall, the manuscript describes a series of carefully executed experiments demonstrating the MP-nsEM equivalency using data obtained for several protein systems. Authors also examine MP performance in workflows involving chemical crosslinking, multi-step purification, and selection of optimal buffer conditions. The ability of MP to provide detailed information on the composition of macromolecular assemblies has been reported previously (Young et al. Science 2018, Malay et al. Nature 2019). Nevertheless, additional examples presented in the current manuscript effectively demonstrate the potential of MP for the quantification of sample heterogeneity. This data should be of interest for laboratories looking to incorporate MP into structural biology workflows and warrants the publication of this manuscript, provided that the authors address the following minor suggestions to further improve the manuscript:

Authors should indicate how the errors listed throughout the manuscript were calculated.

L. 54: SEC-MALS is routinely used to obtain information on both stokes radii and molecular weights—albeit with less resolution than that demonstrated by MP. The authors could consider including a brief note comparing the SEC-MALS and MP capabilities for the benefit of readers familiar with the former method.

L. 70: The authors state that “the principle of operation of MP is remarkably similar to that of nsEM”. From the description of the MP technique that follows that statement, it is clear that the basic principles of both methods are very different with the exception of a surface being involved at one stage of the sample detection process. This might be confusing to some readers. The authors should consider revising this statement.

L. 101: The authors use very interesting data obtained for the trimeric sub-complex of cohesin to demonstrate how MP can be effectively used to obtain mass heterogeneity information for a complex that exhibits structural heterogeneity. This is possible since MP does not seem to be sensitive to the shape distribution heterogeneity and flexibility that can be visualized by EM (Fig. 1 d-e). The authors should consider clarifying this in the text.

L. 211: In the Conclusions section, authors list artefacts and limitations of the nsEM, but not MP. Since MP is a recently developed technique, the authors should include a complete list of its known artefacts and limitations (if any). This would help readers to assess the applicability of MP to their laboratory workflows and, more importantly, better understand data published using this technique.

L. 356/Fig. 2f The comparison of the assembled fractions obtained by MP and nsEM shows a systematic difference for most of the samples. Could the authors comment on the MP tendency to overestimate those values?

L. 368/Fig. 3c,d In the MP distributions, the Ecm29 containing species seem to be unresolved for NaCl concentrations above 100 mM. Were the 26S and 26S+Ecm29 species for high-salt samples fit with separate peaks, or are they indicated on Fig. 3c for orientation only?

L. 400 & 416: When describing the movie fitting parameters, the authors introduce threshold 1 as

related to the particle contrast relative to the background noise. The authors also include a table showing a number of binned frames and threshold 1 values used for different protein systems. Can the authors comment on how the selection of different values of those parameters will affect the MP results in general? Is there a specific relationship between the contrast to noise ratio and the appropriate values of those parameters? More specifically, will the choice of those parameters affect the quantitative results for the systems studied here?

L. 422 Calibration procedure. Can the authors provide more information on the calibration procedure? Specifically: Are the oligomer solutions used for the calibrations related to the protein systems under study? From the molecular weights listed, they seem to be oligomers of two different proteins. If this is the case, what are the expected populations of different oligomeric states, and how will the population differences affect the calibration error? To help readers assess this, the authors should modify supplementary Figure 2 to include both the error bars of contrast values and the fit residuals.

L. 430 Extraction of mole fractions. To obtain mole fractions values, the experimental data were smoothed by kernel density estimation (KDE) and fitted with Gaussian components. This might introduce errors to the final values that are dependent on the chosen KDE bandwidth, and on the treatment of the Gaussian fitting parameters. Can the authors elaborate on what criteria were used to select the KDA bandwidth? How will the selection of those parameters (bandwidth, standard deviations and positions) affect the mole fraction errors?

L. 460 Correction for surface-solution concentrations discrepancies. In the reference cited by the authors (Young et al. 2018), both the increase and decrease of the decay rate with molecular weight was observed for different proteins. What controls did authors perform to confirm that the species described in this manuscript do not exhibit similar behavior?

L. 629 Sample preparation under different salt concentrations/nucleotide conditions. Were the additional proteins included in those samples (apyrase, hexokinase) identifiable in the MP data?

Supplementary Figure 1: The PSF fit residuals (panel c, f) seem to show some systematic features, especially for the lower SNR case. Can the authors comment on how this will affect the fitted MP contrast data?

Supplementary Figure 4: The reproducibility of the MP measurements presented in the manuscript is generally very good. In most cases, the low MW peaks show the most variability (see Supplementary Figure 4, panel a). Can authors comment on the sources of this variability?

Supplementary Figure 9: In comparison with other systems, the KDE bandwidth value used to obtain this plot seems to result in a noisier distribution. Why wasn’t a larger bandwidth value used (as applied to the analysis of the proteasome data)? Additionally, some peaks on this, and earlier plots were omitted in the Gaussian fits. What was the effect of omitting some of the distribution peaks on the final fitted mole fractions? This is particularly relevant to data presented in Supplementary Figure 15, where the high molecular weight peaks are poorly resolved, but the peak’s shoulder above 2.5 MDa was excluded from the analysis.

Reviewer #2 (Remarks to the Author):

The manuscript from Sonn-Segev and colleagues described using mass photometry (MP) to accurately report on sample heterogeneity. Mass photometry is a new technique that has only been introduced very recently. The method itself has certain technique advantage, such as using very little sample, and provide rapidly information on mass of molecules in the sample. In this manuscript, authors attempted to quantifying the sample heterogeneity, using negative stain EM as a control to validate the results obtained from mass photometry. Indeed, MP can provide valuable information regarding

size distributions of molecules presented in the sample. In a way, it is somewhat similar as size exclusion chromatography, although for the purpose of only characterizing the sample but not purify the sample. The approaches described in this manuscript is very interesting and potentially very impactful. It provides real case examples to show that mass photometry can provide valuable information that can facilitate optimizing sample preparation. I can imagine it will become a very popular and easily accessible method.

Despite my enthusiasm about the technique presented in the manuscript, I found the manuscript is very hard to read. While the introduction outlined the importance of characterizing sample heterogeneity, and the brief introductory of mass photometry, I could not figure this out what is the main message the manuscript wants to present. It is not to describe the technique itself, as the basic principle of the technique has been described in the past, ref 16 – 19.

The goal of this manuscript seems to demonstrate its applicability with a few real cases. These real cases are very interesting, and represent a few applications of the method. To prove the method is a quantitative method, author needs to prove further the accuracy of quantifications. From the data presented, it seems that besides molecular weight, other information, such as heterogeneity of sample (in terms of size), is rather qualitative. The word "Quantifying" in the title is misleading, as the examples presented are not quantified information, except molecular weight, which can be determined accurately by other means, and it is not the related to the heterogeneity. "Characterizing" is probably a better word. If the accuracy of the quantification can be established, the mass photometry can be used to explore a lot more quantitative information about the sample, which unfortunately is not shown in the manuscript.

Figure 1c shows one negative stain EM image with individual particles marked. A more appropriate method is to collect a negative stain image dataset, pick particles and run a 2D classifications of particles. That would give a more comprehensive and possibly quantitative comparison. For example, assuming that the total number of events under each peak (integration of the peak) correlates to the relative percentage of particles (is this true?), authors could compare the number of particles in each 2D class of negative stain EM with the corresponding peaks in Figure 1b to quantify that the MP can also provide the relative ratio of different types of particles in the solution. Such quantitative measurement could potentially lead to interesting applications.

Minor:

Introduction: While negative stain EM is often used to evaluate sample homogeneity, it is really a popular but not standard method.

Yifan Cheng

Reviewer #3 (Remarks to the Author):

Sonn-Segev et al. perform a tour-de-force in analyzing a large number of complexes that exhibit heterogeneity by mass photometry. Using their method, they convincingly show that subcomplexes can be identified and that the success of purification procedures and/or cross-linking can be easily and quickly tracked. It is an important study and would be interesting to a variety of readers. I support publication, provided the authors address the following concerns.

Major comments:
1. The description of the instrument and data analysis could be expanded. As it stands, it is difficult for a general reader to understand what is being used for their study.
2. What are their standards for the instrument or data analysis that allow accurate mass determinations? How is this done? There is only one sentence in the manuscript and reference to a

Supp. Fig. This should be expanded upon in the main text, and it may be worthwhile to put it into Main Fig. 1. Furthermore, Fig. S2 does not have labels for proteins in the calibration curve.
3. How are the EM particles converted into contrast signals to compare with the image analysis performed using their technique? This should be explicitly stated in the main text.
4. Cohesin is a cool machine in that in adopts a variety of states. Can the authors comment on how they are able to still get such a homogenous peak for the mass even with this conformational heterogeneity?
5. For Fig. 2g, it would be informative to place the reprojection of a particular subcomplex or fully assembled complex next to the experimental class averages for comparison. This would make the figure extremely powerful. It would also be helpful to discuss that the particles are in characteristic views (or preferred orientations) in this Figure. Non-expert readers may wonder why they all look the same. In fact, this could be one or the arguments for why this technique is so useful. You may not have sufficient views in negative stain EM to tell if you have a certain complex or not.
6. The authors should explain their basis for assuming that the complexes they observe with the proteasome either do or do not contain Ecm29. I don't think the mass is described in the text or table.

Minor comments:
1. Abstract: minimal volume? Isn’t it more important to use a minimal concentration?
2. Negative stain EM is not necessarily slow. My lab can screen many, many samples in one or two hours. It may be slow compared to the author’s technique, but caution should be used in describing speed.
3. line 66: while I agree native MS has associated experimental complexity, it is worth pointing out that it can provide information that the authors' technique cannot, such as the exact identity and stoichiometry of subunits. Work by e.g. Carol Robinson also show how native MS can be used for structural analysis of protein complexes.
4. I’m not convinced that false particle picking is a major concern for negative stain EM. There is plenty of contrast in these images. I’d remove that part.

David Taylor

Reviewers' comments:

Reviewer #1 (Remarks to the Author):

In this manuscript, Sonn-Segev et al. show how the recent label-free method, termed mass photometry (MP), provides information on sample heterogeneity largely equivalent to that obtained by negative stain electron microscopy (nsEM). Since MP data can be collected faster than nsEM, this approach could be of interest to researchers in the field of cryo-electron microscopy and x-ray crystallography.

Overall, the manuscript describes a series of carefully executed experiments demonstrating the MP-nsEM equivalency using data obtained for several protein systems. Authors also examine MP performance in workflows involving chemical crosslinking, multi-step purification, and selection of optimal buffer conditions. The ability of MP to provide detailed information on the composition of macromolecular assemblies has been reported previously (Young et al. Science 2018, Malay et al. Nature 2019). Nevertheless, additional examples presented in the current manuscript effectively demonstrate the potential of MP for the quantification of sample heterogeneity. This data should be of interest for laboratories looking to incorporate MP into structural biology workflows and warrants the publication of this manuscript, provided that the authors address the following minor suggestions to further improve the manuscript:

Authors should indicate how the errors listed throughout the manuscript were calculated.

The mole fraction error calculations are written in the method section under corresponding sub-section (L.365). Errors related to contrast-to-mass calibration are described in the methods in the calibration sub-section (L. 326).

L. 54: SEC-MALS is routinely used to obtain information on both stokes radii and molecular weights—albeit with less resolution than that demonstrated by MP. The authors could consider including a brief note comparing the SEC-MALS and MP capabilities for the benefit of readers familiar with the former method.

We have added the following (two) statements to include this information:

(L. 57) A combination of multi angle light scattering with chromatography (SEC-MALS) is a commonly used technique to determine both Stokes radii and molecular weights, but does not operate under equilibrium conditions and heavily depends on the chromatographic resolution achievable and the accuracy of associated protein quantification by UV absorption.

(L. 238) In comparison to SEC-MALS, MP provides improved resolution, in situ measurement of mixtures without need for separation by chromatography, and much lower demands in terms of sample volume and concentration.

(R1C1)

L. 70: The authors state that “the principle of operation of MP is remarkably similar to that of nsEM”. From the description of the MP technique that follows that statement, it is clear that the basic principles of both methods are very different with the exception of a surface being

involved at one stage of the sample detection process. This might be confusing to some readers. The authors should consider revising this statement.

We agree with the referee that our wording is indeed confusing. We were merely trying to convey the point that no additional sample preparation/manipulation is needed.

We changed the respective section to:

(L. 81) The principle of operation of MP is remarkably similar to the first step of nsEM (Fig. 1a), where placement of a small amount and concentration (<10 µl, <100 nM) of sample onto a substrate leads to non-specific adsorption at a solid-solute interface.

(R1C2)

L. 101: The authors use very interesting data obtained for the trimeric sub-complex of cohesin to demonstrate how MP can be effectively used to obtain mass heterogeneity information for a complex that exhibits structural heterogeneity. This is possible since MP does not seem to be sensitive to the shape distribution heterogeneity and flexibility that can be visualized by EM (Fig. 1 d-e). The authors should consider clarifying this in the text.

We have added the following sentence for clarification:

(L.250) At this point of development, it is unclear to which degree the overall structure of macromolecules well below the diffraction limit affects their quantification by MP. In our previous work [Young] we could not find significant effects of even large structural changes on the measured mass. Evidences so far suggest that MP is insensitive to conformation within the 5-10% level of measured mass, but there is ample scope for future exploration of these details in the future.

(R1C3)

L. 211: In the Conclusions section, authors list artefacts and limitations of the nsEM, but not MP. Since MP is a recently developed technique, the authors should include a complete list of its known artefacts and limitations (if any). This would help readers to assess the applicability of MP to their laboratory workflows and, more importantly, better understand data published using this technique.

We have added the following section as requested:

(L.241) The main limitations of MP, on the other hand, are a lack of structural detail, as well as a currently limited concentration range (<100 nM) due to the single particle nature of the approach. We expect the concentration limitations to be lifted in the near future with improvements in assays, instrumentation, and data analysis. We expect these concentration limitations to be lifted in the near future due to improvements in assays, instrumentation and data analysis. We also emphasise that measurements at lower concentrations are still valuable for two reasons: (1) As long as the associated off-rate is on the order of minutes or slower, the distributions obtained by MP are representative of those at higher concentrations. (2) Even in the case of faster off-rates, comparison of different samples with MP will be valuable, unless very fast off-rates on the order of seconds are encountered, because complexes will not have completely dissociated prior to measurement.

(R1C4)

L. 356/Fig. 2f The comparison of the assembled fractions obtained by MP and nsEM shows a systematic difference for most of the samples. Could the authors comment on the MP tendency to overestimate those values?

We have added the following clarification:

(L.189) The slight, but systematic difference between predicted assembled fraction by MP as compared to nsEM could potentially be due to the different ways mole fractions were calculated in the MP and nsEM (See methods), with MP quantifying only the four (sub-) complexes illustrated in **Fig. 2d** (See Methods for detailed description), while nsEM picking the full APC/C complex out of all detected particles. At the same time, however, we cannot exclude more fundamental differences due to sample behavior, particulary in negative stain where problems such as preferred orientations arise.

(R1C5)

L. 368/Fig. 3c,d In the MP distributions, the Ecm29 containing species seem to be unresolved for NaCl concentrations above 100 mM. Were the 26S and 26S+Ecm29 species for high-salt samples fit with separate peaks, or are they indicated on Fig. 3c for orientation only?

The referee is entirely correct. We used to low salt distributions to obtain information on peak position and peak shape, and then used it for higher salt to estimate the much smaller contributions of 26S+Ecm29. We have now clarified this by adding information:

(L.357) For the evaluation of the relative contributions of 26S and 26S+Ecm29 species in Figure 3c, we used peak information from the low salt spectra (0, 50 mM), and fixed peak position and width for the higher salt spectra, where the different species could no longer be clearly resolved.

(R1C6)

L. 400 & 416: When describing the movie fitting parameters, the authors introduce threshold 1 as related to the particle contrast relative to the background noise. The authors also include a table showing a number of binned frames and threshold 1 values used for different protein systems. Can the authors comment on how the selection of different values of those parameters will affect the MP results in general? Is there a specific relationship between the contrast to noise ratio and the appropriate values of those parameters? More specifically, will the choice of those parameters affect the quantitative results for the systems studied here?

We thank the referee for this comment, We have added the following section, to clarify why those parameters are chosen. Following the referee comment we reviewed the fitting parameters for Trimeric cohesin and changed them to standard values (nf=5, thershold 1=0.8), followed by changes to the associated Figures (Fig.1d and Supplementary Fig. 3b) and Table 1.

(L. 309) The standard values of binned frames (nf) are between 3-5 frames, while Threshold 1 values varies according to the contrast to noise values, with standard values between 0.5-1.5. To quantify low mass proteins below 100-200 kDa either lower Threshold 1, and or higher nf values are chosen (such as in NPC). In cases where the samples have

noisier backgrounds, usually related to buffer content or high amounts of smaller proteins, Threshold 1 is increased to 2 or 3, to avoid detection of low mass signals which are not quantitative in those cases. For APC/C and proteasome samples, the values of nf (3-5) and Threshold 1 (2-3.5) are almost equivalent, since changes in those values will affect only the low mass regime (< 300 kDa). Therefore, all masses below 300 kDa in APC/C and proteasome samples are not used for the quantitative analysis of mole fractions.

(R1C7)

L. 422 Calibration procedure. Can the authors provide more information on the calibration procedure? Specifically: Are the oligomer solutions used for the calibrations related to the protein systems under study? From the molecular weights listed, they seem to be oligomers of two different proteins. If this is the case, what are the expected populations of different oligomeric states, and how will the population differences affect the calibration error? To help readers assess this, the authors should modify supplementary Figure 2 to include both the error bars of contrast values and the fit residuals.

We have added the following section and associated Supplementary Figure 2 to provide the information requested by the referee.

(L.329) The mass distributions for the two protein oligomer solutions are shown in Supplementary Fig. 2b and c. The achievable precision of the order of 2% of the object mass for repeated measurements, which is intrinsic to mass photometry, is larger than the standard error of the mean $\sigma/N^{1/2}$, given that σ < 20 kDa throughout the mass range used, and N is >100.

(R1C8)

L. 430 Extraction of mole fractions. To obtain mole fractions values, the experimental data were smoothed by kernel density estimation (KDE) and fitted with Gaussian components. This might introduce errors to the final values that are dependent on the chosen KDE bandwidth, and on the treatment of the Gaussian fitting parameters. Can the authors elaborate on what criteria were used to select the KDA bandwidth? How will the selection of those parameters (bandwidth, standard deviations and positions) affect the mole fraction errors?

The choice of KDE bandwidth is usually made taking into account both the experimental capabilities in terms of mass resolution vs mass, and sample-specific features – in other words, we use the largest bandwidth possible without significantly affecting the bandwidth of major mass features. The position of the peaks is determined by local maximum in the approximate neighbourhood of each known species (with the prior knowledge on expect mass), except for high salt concentrations in the proteasome analysis, where the peak position was fixed based on low salt cases. We examined the sensitivity of different bandwidth (within a reasonable range) to the spread of the mole fraction and found it to be mostly smaller than the spread of mole fraction in different repeats. We added the following sentence clarifying that in methods:

(L. 365) Extracting the mole fraction from KDEs generated with different bandwidths resulted in minor differences in the mole fractions values, smaller than the differences between experimental repeats, therefore mole fraction errors were calculated based on the spread obtained from repeats.

(R1C9)

L. 460 Correction for surface-solution concentrations discrepancies. In the reference cited by the authors (Young et al. 2018), both the increase and decrease of the decay rate with molecular weight was observed for different proteins. What controls did authors perform to confirm that the species described in this manuscript do not exhibit similar behavior?

We thank the referee for this comment. In contrast to Young et al, in our work the examined species have overlapping mass distributions, which limits our ability to quantify the decay in landing rate for each species separately. As a first approximation, we attributed molecular weight contributions as the main source of discrepancies between different species, i.e., all species have the same affinity to the glass-water interface, since this seemed to be the dominant factor in previous work (Young et al.). We took a weighted average approach to calculate the decay rate and proportionality prefactor (α), which turned out to be similar to the value obtained by Young et al. (0.2-0.3 vs 0.3), supporting our approximation. In addition, based on our previous work, the unexpected behavior of increase in decay rate with higher oligomers was observed only in two proteins, while the common decreasing behavior was observed in several systems. Specifically, only for alcohol dehydrogenase and β-amylase, which are predominantly tetramers, was this observed. In those cases, the tetrameric species exhibited decay rates consistent with the described molecular weight trend across the other samples measured, and it was only the dissociated dimer that showed the unexpected behaviour. Still, we cannot exclude the possibility that factors other than diffusion contribute to the binding rate of different species to the glass. Importantly, this correction changed the mole fraction values by only a small percentage (≤3%) as presented in Supplementary Tables 2 and 4.

We have amended the description in the Methods section to clarify these assumptions and more clearly explain the approach we took, starting at L. 381

(R1C10)

L. 629 Sample preparation under different salt concentrations/nucleotide conditions. Were the additional proteins included in those samples (apyrase, hexokinase) identifiable in the MP data?

We could not identify the signal from Apyrase (45 kDa) or hexokinase (100 kDa). The reason is that the proteasome samples had a noisy background due to an imperfect buffer. We therefore set the threshold such that small protein/ background (<200-250kDa) were ignored. We have added the following statement for clarification:

(L. 570) We could not detect either Apyrase or hexokinase at the detection conditions used, which were optimised to suppress buffer background and therefore limited detection to >200 kDa species.

(R1C11)

Supplementary Figure 1: The PSF fit residuals (panel c, f) seem to show some systematic features, especially for the lower SNR case. Can the authors comment on how this will affect the fitted MP contrast data?

We believe that the referee is referring to the non-shot noise residuals after fitting, which is a consequence of using a model point spread function that does not capture all features of the experimental one. We have generally found that such residuals have little effect on the obtained signal magnitude as long as the same PSF is applied throughout. We have now added a respective statement after the relevant section

(L. 305) Our model PSF does not necessarily capture the spatially more extending features of our PSF, which means that the fit residuals are not entirely dominated by shot noise (Supplementary Figure 1c,f). Based on experience, we find that these differences do not introduce a systematic error in quantification of the signal, as long as the same PSF model is applied for the entire data set and calibration.

(R1C12)

Supplementary Figure 4: The reproducibility of the MP measurements presented in the manuscript is generally very good. In most cases, the low MW peaks show the most variability (see Supplementary Figure 4, panel a). Can authors comment on the sources of this variability?

The source of this variability is a consequence of non-unity detection efficiency at low mass for the analysis parameters used, and non-ideal buffer conditions (discussed above in respect to the fitting parameters). We have added a clarifying statement to the caption of Supplementary Figure 4a.

The observed increased variability at low compared to high mass is a consequence of non-unity detection efficiency for the detection parameters used.

(R1C13)

Supplementary Figure 9: In comparison with other systems, the KDE bandwidth value used to obtain this plot seems to result in a noisier distribution. Why wasn't a larger bandwidth value used (as applied to the analysis of the proteasome data)? Additionally, some peaks on this, and earlier plots were omitted in the Gaussian fits. What was the effect of omitting some of the distribution peaks on the final fitted mole fractions? This is particularly relevant to data presented in Supplementary Figure 15, where the high molecular weight peaks are poorly resolved, but the peak's shoulder above 2.5 MDa was excluded from the analysis.

As mentioned above, the choice of KDE bandwidth is usually made taking into account both the experimental capabilities in terms of mass resolution vs mass, and sample-specific features The limited choice of fitting parameters was based on an attempt to not overfit the spectra and focus and quantify features that we independently knew were present. We have now clarified these aspects in the respective figure captions:

For SI Fig9:

The KDE bandwidth (20 kDa) was chosen as a compromise between the known mass resolution of the system and avoiding obvious broadening of the mass distribution as a consequence of applying the KDE. We limited the fit to the four main spectral features to avoid overfitting.

For SI Fig15:

KDE bandwidths and number of fitting parameters was chosen as discussed in Supplementary Figure 9.

(R1C14)

Reviewer #2 (Remarks to the Author):

The manuscript from Sonn-Segev and colleagues described using mass photometry (MP) to accurately report on sample heterogeneity. Mass photometry is a new technique that has only been introduced very recently. The method itself has certain technique advantage, such as using very little sample, and provide rapidly information on mass of molecules in the sample. In this manuscript, authors attempted to quantifying the sample heterogeneity, using negative stain EM as a control to validate the results obtained from mass photometry. Indeed, MP can provide valuable information regarding size distributions of molecules presented in the sample. In a way, it is somewhat similar as size exclusion chromatography, although for the purpose of only characterizing the sample but not purify the sample. The approaches described in this manuscript is very interesting and potentially very impactful. It provides real case examples to show that mass photometry can provide valuable information that can
facilitate optimizing sample preparation. I can imagine it will become a very popular and easily accessible method.

Despite my enthusiasm about the technique presented in the manuscript, I found the manuscript is very hard to read. While the introduction outlined the importance of characterizing sample heterogeneity, and the brief introductory of mass photometry, I could not figure this out what is the main message the manuscript wants to present. It is not to describe the technique itself, as the basic principle of the technique has been described in the past, ref 16 – 19.

The goal of this manuscript seems to demonstrate its applicability with a few real cases. These real cases are very interesting, and represent a few applications of the method. To prove the method is a quantitative method, author needs to prove further the accuracy of quantifications. From the data presented, it seems that besides molecular weight, other information, such as heterogeneity of sample (in terms of size), is rather qualitative. The word “Quantifying” in the title is misleading, as the examples presented are not quantified information, except molecular weight, which can be determined accurately by other means, and it is not the related to the heterogeneity. “Characterizing” is probably a better word. If the accuracy of the quantification can be established, the mass photometry can be used to explore a lot more quantitative information about the sample, which unfortunately is not shown in the manuscript.

Figure 1c shows one negative stain EM image with individual particles marked. A more appropriate method is to collect a negative stain image dataset, pick particles and run a 2D classifications of particles. That would give a more comprehensive and possibly quantitative comparison. For example, assuming that the total number of events under each peak (integration of the peak) correlates to the relative percentage of particles (is this true?), authors could compare the number of particles in each 2D class of negative stain EM with the corresponding peaks in Figure 1b to quantify that the MP can also provide the relative ratio of different types of particles in the solution. Such quantitative measurement could potentially lead to interesting applications.

We regret the lack of clarity – however, we believe that we have done exactly what the referee suggests in Figure 2 in much detail, and also in Figure 3. Figure 1 was intended to simply introduce the principle and provide a qualitative understanding of the method and how it compares with EM in principle. All the quantification was done in Figure 2, and more was done in Figure 3. Given the comments from the other referees, we would suggest to keep the manuscript as is in this context. At the same time, we refer the referee to the significant changes, clarifications and additions in response to the comments by the referees.

Minor:

Introduction: While negative stain EM is often used to evaluate sample homogeneity, it is really a popular but not standard method.

We have changed the wording accordingly to ‘popular’ in both abstract and introduction.

(R2C1)

Yifan Cheng

Reviewer #3 (Remarks to the Author):

Sonn-Segev et al. perform a tour-de-force in analyzing a large number of complexes that exhibit heterogeneity by mass photometry. Using their method, they convincingly show that subcomplexes can be identified and that the success of purification procedures and/or cross-linking can be easily and quickly tracked. It is an important study and would be interesting to a variety of readers. I support publication, provided the authors address the following concerns.

Major comments:
1. The description of the instrument and data analysis could be expanded. As it stands, it is difficult for a general reader to understand what is being used for their study.

Given that we used a commercial instrument, providing a detailed description beyond the model and manufacturer is difficult as these details are proprietary to the instrument manufacturer. The same is true given that we used commercial MP software, which we tried to address by giving details of the analysis parameters. Given these two sets of information, any user should be able to reproduce the experiments and analysis reported here, subject to any sample variability. To nevertheless provide more detail, we added a statement with respect to the nature of the instrument.

(L.280) ...which were performed on a home-built mass photometer constructed as discussed in detail by Cole et al., except for operation at the same wavelength as the commercial instrument (520 nm).

(R3C1)

2. What are their standards for the instrument or data analysis that allow accurate mass determinations? How is this done? There is only one sentence in the manuscript and reference to a Supp. Fig. This should be expanded upon in the main text, and it may be worthwhile to put it into Main Fig. 1. Furthermore, Fig. S2 does not have labels for proteins in the calibration curve.

The reason we kept this section short is because the procedure and associated accuracy is described in much detail in the cited reference (Young et al. Science 2018). We have now expanded the corresponding Supplementary Figure, including the corresponding mass distributions and error bars. We identified the respective masses. Ultimately, as shown in Young et al., the protein identity is irrelevant, one simply requires a few known masses over the 0-1MDa range. We have clarified these aspects in the caption of the respective supplementary figure. The caption now reads:

Supplementary Figure 2: Contrast-to-mass (C2M) calibration curve. a, The contrasts of two proteins of known mass with different oligomeric states (Protein 1: 90, 180, 360, 540 kDa - red; Protein 2: 66, 132 and 198 kDa – green) are plotted vs their known mass. The black line is the fit to the data according to y=bx, with b representing the C2M calibration factor. The fitting residuals are presented in the above panel as a percentage mass error. The contrast distributions of Protein 1 (b) and Protein 2 (c) exhibit the relative abundances of the different oligomers. The numbers above each correspond to their mass in kDa. In principle, any mixture or combination of proteins with known mass can be used for mass calibration. We chose oligomeric proteins because they provide multiple reference points in a single measurement, thereby accelerating and simplifying the process.

(R3C2)

3. How are the EM particles converted into contrast signals to compare with the image analysis performed using their technique? This should be explicitly stated in the main text.

We are afraid that this is a misunderstanding. We do not convert EM particles into contrast signals. We used standard image processing including classification and averaging to identify the subcomplex each particle represents and assigned a mass accordingly.

4. Cohesin is a cool machine in that in adopts a variety of states. Can the authors comment on how they are able to still get such a homogenous peak for the mass even with this conformational heterogeneity?

As explored in our original publication (Young et al), mass photometry appears to be insensitive to conformational changes occurring on the sub-diffraction level within the error of the current measurement. As a result, complex structure makes no measurable difference for our mass measurement at this stage of the development of the technique. We now added a sentence clarifying this aspect

(L.119) This monodispersity of the peak is enabled by the apparent insensitivity of MP signals to large scale structural variability on the sub-diffraction length scale, as demonstrated with myosin IIb in our original study [Young].

(R3C3)

5. For Fig. 2g, it would be informative to place the reprojection of a particular subcomplex or

fully assembled complex next to the experimental class averages for comparison. This would make the figure extremely powerful. It would also be helpful to discuss that the particles are in characteristic views (or preferred orientations) in this Figure. Non-expert readers may wonder why they all look the same. In fact, this could be one or the arguments for why this technique is so useful. You may not have sufficient views in negative stain EM to tell if you have a certain complex or not.

We added the requested reprojections to figure 2.

(R3C3)

6. The authors should explain their basis for assuming that the complexes they observe with the proteasome either do or do not contain Ecm29. I don't think the mass is described in the text or table.

We routinely submit our preparation to mass spectrometric identification and found five known proteasome interactors in the preparation as shown in Figure 3a. Most significantly we see an almost stociometric band corresponding to ECM29 as most abundant of these interactors. The referee is absolutely correct that we did not mention this in the text explicitly. It has a mass of 200 kDa. We now added the mass to L. 205.

(R3C4)

Minor comments:
1. Abstract: minimal volume? Isn't it more important to use a minimal concentration?

We would prefer to keep the statement as is. The reason is that using little sample does have advantages compared to other bulk methods (e.g. DLS). Low concentrations could be misunderstood in the context that cryoEM usually operates in the high nM to low µM regime.

2. Negative stain EM is not necessarily slow. My lab can screen many, many samples in one or two hours. It may be slow compared to the author's technique, but caution should be used in describing speed.

We do agree that initial screens for quality control using negative stain can indeed be quick, however a substantially informative analysis of the sample requires data collection and processing that may take up to hours or days depending on the efficiency of the data analysis workflow. We have further clarified this:

(L. 68) While the molecular detail that can be extracted is often helpful for further study at high resolution, data processing and analysis workflows can range from hours to several days, making high-throughput screening impractical.

(R3C5)

3. line 66: while I agree native MS has associated experimental complexity, it is worth pointing out that it can provide information that the authors' technique cannot, such as the exact identity and stoichiometry of subunits. Work by e.g. Carol Robinson also show how native MS can be used for structural analysis of protein complexes.

We have amended the statement as requested to minimise misunderstandings to:

(L. 75) Advances in native mass spectrometry (MS) over the past decades[10–12] have allowed for much higher mass resolution and can be used for structural analysis, but the associated experimental complexity and non-native conditions have prevented native MS from becoming a widely used tool in this context.

We believe that it is still fair to say that native MS is not as widely used as DLS or SEC-MALS for example because it is more difficult to operate than solution-based methods, MP being one of them.

(R3C6)

4. I'm not convinced that false particle picking is a major concern for negative stain EM. There is plenty of contrast in these images. I'd remove that part.

We do have to disagree here with the reviewer. While indeed the contrast is much better and indeed globular particles can be picked without many false picks in negative stain, picking of heterogeneous datasets as we are doing here is still a challenge even for the most modern picking tools and false negative as well as false positive picks as well as double picks of the same particle do occur in significant amounts and compromises have to be made. We would thus prefer to keep the statement as is.

David Taylor
